# Model constructions of chemosensitivity and prognosis of high grade serous ovarian cancer based on evaluation of immune microenvironment and immune response

**DOI:** 10.1186/s12935-021-02295-y

**Published:** 2021-11-04

**Authors:** Han Zhang, Yijun Wu, Hao Li, Liping Sun, Xiangkai Meng

**Affiliations:** 1 Department of Gynecology, The First Hospital of China Medical University, Shenyang, Liaoning People’s Republic of China; 2 Tumor Etiology and Screening Department of Cancer Institute and General Surgery, Key Laboratory of Cancer Etiology and Prevention in Liaoning Education Department, and Key Laboratory of Gastrointestinal Cancer Etiology and Prevention in Liaoning Province, The First Hospital of China Medical University, Shenyang, Liaoning People’s Republic of China; 3 Department of Clinical Laboratory, The First Affiliated Hospital of China Medical University, Shenyang, Liaoning People’s Republic of China

**Keywords:** Ovarian cancer, Differentially expressed genes, Prognosis, Immune, Chemosensitivity

## Abstract

**Background:**

The prognosis of high grade serous ovarian cancer (HGSOC) patients is closely related to the immune microenvironment and immune response. Based on this, the purpose of this study was to construct a model to predict chemosensitivity and prognosis, and provide novel biomarkers for immunotherapy and prognosis evaluation of HGSOC.

**Methods:**

GSE40595 (38 samples), GSE18520 (63 samples), GSE26712 (195 samples), TCGA (321 samples) and GTEx (88 samples) were integrated to screen differential expressed genes (DEGs) of HGSOC. The prognosis related DEGs (DEPGs) were screened through overall survival analysis. The DEGs-encoded protein–protein interaction network was constructed and hub genes of DEPGs (DEPHGs) were generated by STRING. Immune characteristics of the samples were judged by ssGSEA, ESTIMATE and CYBERSORT. TIMER was used to analyze the relationship between DEPHGs and tumor-infiltrating immunocytes, as well as the immune checkpoint genes, finally immune-related DEPHGs (IDEPHGs) were determined, and whose expression in 12 pairs of HGSOC tissues and tumor-adjacent tissues were analyzed by histological verification. Furthermore, the chemosensitivity genes in IDEPHGs were screened according to GSE15622 (n = 65). Finally, two prediction models of paclitaxel sensitivity score (PTX score) and carboplatin sensitivity score (CBP score) were constructed by lasso algorithm. The area under curve was calculated to estimate the accuracy of candidate gene models in evaluating chemotherapy sensitivity.

**Results:**

491 DEGs were screened and 37 DEGs were identified as DEPGs, and 11 DEPHGs were further identified. Among them, CXCL13, IDO1, PI3, SPP1 and TRIM22 were screened as IDEPHGs and verified in the human tissues. Further analysis showed that IDO1, PI3 and TRIM22 could independently affect the chemotherapy sensitivity of HGSOC patients. The PTX score was significantly better than TRIM22, PI3, SPP1, IDO1 and CXCL13 in predicting paclitaxel sensitivity, so was CBP score in predicting carboplatin sensitivity. What’s more, both of the HGSOC patients with high PTX score or high CBP score had longer survival time.

**Conclusions:**

Five IDEPHGs identified through comprehensive bioinformatics analysis were closely related with the prognosis, immune microenvironment and chemotherapy sensitivity of HGSOC. Two prediction models based on IDEPHGs might have potential application of chemotherapy sensitivity and prognosis for patients with HGSOC.

**Supplementary Information:**

The online version contains supplementary material available at 10.1186/s12935-021-02295-y.

## Introduction

Ovarian cancer (OC) is a common gynecological malignancy, The 5-year survival rate of OC patients remains low because of the high heterogeneity of the tumor and high incidence of drug resistance during chemotherapy [1–5]. The standard treatment for OC is maximal cytoreductive surgery and postoperative paclitaxel-platinum combined chemotherapy, with a higher survival rate than other regimens, including cyclophosphamide-platinum/gemcitabine and so on [6]. Most patients develop treatment resistance after prolonged treatment, and almost all patients with recurrent ovarian cancer (ROC) eventually develop platinum resistance [7, 8]. But paclitaxel still keeps anti-tumorigenic activity for patients with platinum-resistant OC or platinum-resistant ROC [9]. In recent years, new immunotherapy strategies like immune checkpoint inhibitors therapy, cancer vaccine inoculation and adoptive immunotherapy have had a broad prospect for OC patients with platinum resistance in clinical treatment. In addition, new immunotherapies such as chimeric antigen receptor T cells (CAR-T) and mesothelin (meso) have progressed into clinical trials for the treatment of OC [10].

High grade serous ovarian cancer (HGSOC) is highly malignant and can easily metastasize to the abdominal cavity causing severe ascites and intestinal obstruction as the main type of epithelial OC, which occupies 70%-80% of epithelial ovarian cancer deaths [11]. The clinical manifestations of HGSOC are atypical and lack of specific detection methods, and the five-year survival rate is only 15–25%, so it is urgent to develop precise treatment for HGSOC [12, 13].

Traditionally, clinical stage, the status of cytoreductive surgery and sensitivity to chemotherapeutic drugs are fundamental factors that predict the prognosis of patients with OC [14]. However, because ovarian cancer is a heterogeneous disease characterized by complex molecular and genetic changes, the survival rate and treatment response of cases with similar clinical features are very different, and it is more reasonable to judge the prognosis at the molecular biology level [15]. In recent years, a series of studies has confirmed that the expression level of genes in patients can significantly determine their long-term survival or recurrence after chemotherapy, suggesting that crucial genes can be selected as biomarkers to provide a reference for prognosis prediction and treatment choice of patients [16, 17].

The immune system plays an important regulatory role in the genesis and progression of OC, and the tumor immune reaction is closely correlated with the clinical efficacy and the outcome of OC cases. Eiichi Sato et al. reported that tumor-infiltrating lymphocytes (TILs) in the immune microenvironment of ovarian cancer could assist in tumor cell removal [18]. Additionally, the increase in the ratio of CD8 + /CD4 + T lymphocytes could improve the overall survival rate of patients with OC [19]. Some DEGs in patients could participate in the regulation of immunocyte infiltration in ovarian cancer, leading to immune escape and adverse clinical outcome by promoting the production of cytokines and inhibiting the proliferation of effector T lymphocytes [20].

Activating immune reaction and destroying the proliferation and invasion of OC have become a hot spot in clinical immunotherapy because of the close relationship between OC malignant progression and immune escape mechanism. Immune destruction against tumor is a multi-step and coordinated process, which can take targeted regulation at several key points (immune checkpoints) to induce the tumor rejection. Immune checkpoint inhibitors (ICPIs) can prevent immune escape and induce the immune system to produce an anti-tumor response by blocking the activation of T lymphocytes surface checkpoint proteins, thus improving the efficacy of chemotherapy and effectively prolonging the survival time of tumor cases [21]. Now that the role of ICPIs in ovarian cancer is more moderate and changeable, the immunotherapy of ovarian cancer must comprehensively consider the immune suppressive network in the tumor microenvironment, as well as the inherent biological characteristics of the tumor that also plays a decisive role [22]. Accordingly, searching the differential expressed genes of ovarian cancer with a significant correlation with tumor-infiltrating immunocytes and immune checkpoints, and exploring biomarkers that could predict the responsiveness of patients to different types of immunotherapy are of considerable significance, which contributes to choose the best combination therapy and reduce the side effects [23].

Therefore, this study intends to analyze the association between prognosis-related genes and tumor-infiltrating immunocytes, as well as immune checkpoint genes, in ovarian cancer through bioinformatics methods. Thus, the potential prognostic biomarkers that are closely associated with the tumor immune response and curative effect can be explored, and scoring models can be constructed, providing potential markers for prognosis and chemotherapy sensitivity evaluation, as well as new valid targets to treat OC.

## Materials and methods

### Microarray data

The Gene Expression Omnibus (GEO, https://www.ncbi.nlm.nih.gov/geo/) includes high-throughput sequencing data, gene chips, microarrays and other massive data information that users can download for free. We obtained three gene expression profiles (GSE40595, GSE18520 and GSE26712) from the GEO database. Three microarray datasets were integrated after removing batch effect using R-package “sva” (http://www.bioconductor.org.) [24]. Among them, GSE40595 included 32 HGSOC and 6 normal samples, GSE18520 contained 53 HGSOC and 10 normal samples, and GSE26712 included 185 HGSOC and 10 normal samples [25–27].

Besides, we got GSE15622 dataset obtaining the information about chemotherapy sensitivity of OC from GEO database, and it included 36 OC samples taking paclitaxel monotherapy and 29 OC samples taking carboplatin monotherapy [28].

TCGA is a highly reliable high-throughput database containing DNA, RNA and protein information from various human cancers [29]. Sequencing data and clinical information of TCGA-OV 379 OC samples were obtained from UCSC database (http://xenabrowser.net/hub/), and finally 321 HGSOC samples were incorporated after screening according to the clinical information [30]. At the same time, we supplemented the sequencing data of 88 normal ovarian epithelial tissues through GTEx database, and took joint analysis with HGSOC samples from TCGA database [31].

### Data processing

We used “limma” package (http://www.bioconductor.org.) to perform background correction and differential gene analysis respectively on chip data and transcriptome high-throughput sequencing data [32]. We set adj. P < 0.05 and | logFC |> 1 as the cut-off criteria. Thus, we distinguished DEGs between HGSOC and normal samples in GSE40595, GSE18520 and GSE26712, as well as those in TCGA-OV and GTEx datasets. And we took the intersection of the above DEGs for subsequent analysis.

Volcano maps and heat maps were drawn to visualize the results of DEGs analysis using “ggplot2” package (https://ggplot2.tidyverse.org) and “pheatmap” package (https://CRAN.R-project.org/package=pheatmap) [33]. The volcano map took the multiple of gene expression difference as the abscissa, and the logarithm of adj. P value as the ordinate, and can directly reflect the DEGs in two groups of samples through scatter diagram. The heat map reflected the data information in gene expression matrix by the change of color, and took clustering analysis of abundance similarity among samples.

### Survival analysis

We finally obtained 320 HGSOC cases with complete survival information out of 321 HGSOC cases from TCGA-OV database after screening the clinical information. “Survival” package (https://CRAN.R-project.org/package=survival) was used for Kaplan–Meier survival analysis, and we set median single gene expression as the cut-off criteria to divide patients into high gene expression group and low gene expression group. Then we estimated the effect of single gene on overall survival (OS) rate in HGSOC patients and evaluated the survival curve by log-rank test. We set log rank P < 0.05 as the cut-off criteria. In addition, univariate COX regression analysis was used to calculate univariate hazard ratio (HR) through "survival" package, and multivariate COX regression analysis was used to identify independent prognostic factors.

### Clinical specimens collection and histological verification

Twelve pairs of tumor tissues and tumor-adjacent tissues were collected in accordance with the Declaration of Helsinki and legal regulations from HGSOC patients in the First Hospital of China Medical University. Tumor-adjacent tissue was defined as normal ovarian tissue > 5 cm away from the tumor. The study was approved by the Ethics Committee of the First Hospital of China Medical University. The written informed consent has been obtained from each participant before specimen collection.

After crushing and grinding at low temperature, tissue RNA extraction was performed according to the Trizol Reagent (Takara, Japan) protocol. RNA was reversely transcribed into cDNA according to the MonScript™ rtIII All-in-One Mix with dsDNase (Monad, China) protocol. 2 μg total RNA of each sample was added to 40 μL reverse transcription reaction system. The temperature protocol of reverse transcription was as follows: 37 ℃ for 2 min to remove the contamination of genomic DNA, then 55 ℃ for 15 min and 85 ℃ for 5 min. The products were stored at − 20 ℃. Relative mRNA expression levels were detected by real-time quantitative PCR (RT-qPCR) using SYBR^®^ Premix Ex Taq^TM^ II (Takara, Japan).

A standard three-step real-time PCR program was used with an annealing temperature of 58℃ and 40 cycles of amplification. β-actin was selected as the internal reference. All of the RT-qPCR curves were with single peak. The relative quantification in RT-qPCR was calculated by 2^-Δct^ method. P < 0.05 was considered to imply significant results. The sequences of primers were listed in the Additional file 5: Table S5.

### Prognostic PPI network construction and core module identification

The Search Tool for the Retrieval of Interacting Genes (STRING, https://string-db.org/) is an online tool to analyze protein interactions in multiple ways [34]. First, we used the tool to establish the protein–protein interaction (PPI) network of DEGs intersection and search for interacting genes. Then, we visualized the PPI network using MCODE plug-in from Cytoscape software and identified core modules [35]. MCODE’s (molecular complex detection) main function is clustering in protein network and building functional modules, which can find out the closely-associated areas that may represent highly function-related molecular complexes according to the connection of each node in PPI network.

### ssGSEA, ESTIMATE and CYBERSORT

We collected 29 immune related gene sets from published literatures, and among them there were 569 immune related genes involved (Additional file 1: Table S1) [36–38]. “GSVA” package (http://www.biomedcentral.com/1471-2105/14/7) uses single sample gene set enrichment analysis (ssGSEA) to calculate rank value of every gene from expression profiles and quantify the enrichment fraction of each immune related gene in a single sample, which can help judge the activity of immune cells, immune function or immune pathway of each sample [39].

The "ESTIMATE" package (https://R-Forge.R-project.org/projects/estimate/) is also based on the ssGSEA principle to assess the stroma content, the proportion of immune cells and the purity of the tumor in every single sample, which is mutually verified with the results of the "GSVA" package [40].

CYBERSORT is an analytical tool developed by Newman et al., which can deconvolute the expression matrices based on the known reference sets to estimate the abundance of different types of immune cells in the mixed cell population [41]. The expression matrix is analyzed by CYBERSORT through "e1071" package (http://cran.r-project.org/web/packages/e1071/index.html), and the results are visualized through "pheatmap", "vioplot" and "corrplot" packages.

### Correlation analysis among hub gene expression and immunocyte infiltration, as well as immune checkpoints

TIMER (https://cistrome.shinyapps.io/timer/), which can be applied to analyze tumor immune relevance, was used to analyze the correlation between gene expression from high-throughput sequencing dataset of serous ovarian cancer in TCGA-OV and six tumor infiltrating immunocytes (B lymphocytes, CD8 + T lymphocytes, CD4 + T lymphocytes, neutrophils, macrophages and dendritic cells), as well as five immune checkpoint genes (PDCD1, CD274, CTLA4, HAVCR2 and TOX) [42]. Significance levels were set at the 5% level.

Furthermore, CYBERSORT was used to verify the results of TIMER.

### Gene set enrichment analysis (GSEA)

The sequencing data of 321 HGSOC samples from TCGA-OV database were divided into two groups according to the median of single gene expression level. The enrichment scores (ES) of pathway-related gene sets in each group were calculated by GSEA software (4.1.0), and reflected the degree to which a given gene set is represented in a ranked list of genes [43]. We choose adj.P < 0.05 and top 20 signaling pathway rank as threshold.

### Identification of genes related to chemosensitivity of paclitaxel / carboplatin in HGSOC patients

We divided patients from GSE15622 into paclitaxel resistant group, paclitaxel sensitive group, carboplatin resistant group and carboplatin sensitive group according to their clinical information, and respectively extracted the expression levels of single gene (CXCL13, IDO1, PI3, SPP1 and TRIM22) in the expression profile and then screened the DEGs between groups. Finally, we visualized the data using “ggplot2” package (https://ggplot2.tidyverse.org).

### Gene ontology (GO) analysis

GO analysis is widely used in the field of bioinformatics, covering three aspects of biology: cellular components (CC), molecular function (MF), and biological processes (BP). Through GO analysis, it is possible to understand the biological functions of DEGs enrichment. The "limma" package was used to screen the DEGs between groups, the "clusterProfiler" package was used for GO enrichment analysis [44], and the "enrichplot" and "ggplot2" packages were used to visualize the results.

### Construction of PTX score model and CBP score model

We chose GSE15622 dataset to screen genes incorporated in models from CXCL13, IDO1, PI3, SPP1 and TRIM22 using lasso algorithm by “glmnet” package, and calculated the corresponding coefficients [45]. The "glmnet" package uses the cyclic coordinate descent method to achieve the final lasso regression model, and each parameter included is optimized and cycled while keeping the other parameters fixed until the coefficient is stable. Lasso algorithm can filter out variables and optimize the complexity of the model. Variable filtering refers to including variables selectively into the model to get better performance parameters. Complexity adjustment is to adjust the model complexity through changing a series of parameters to avoid overfitting. Finally, the lasso algorithm can simplify the model and get the optimal calculation formula.

Thus, we could get PTX score model and CBP score model from expression profile data and corresponding single gene coefficients, and the formula was as follows:

$$Score = \mathop \sum \limits_{i = 1}^{n} Coef\left( i \right)X\left( i \right)$$

N is the number of included genes, Coef(i) is the coefficient, X(i) is the gene expression level.

### Evaluation of the chemotherapy sensitivity of PTX score and CBP score

Firstly, we compared the differences of PTX score between paclitaxel resistant group and paclitaxel sensitive group, and differences of CBP score between carboplatin resistant group and carboplatin sensitive group according to the clinical information. Secondly, we divided patients into groups based on the median value of PTX score or CBP score, and calculated the percentages of drug-resistant and drug-sensitive groups alone. Finally, we used GraphPad Prism 8 software to draw and visualize ROC curve, calculated the area under curve (AUC) and estimated the accuracy of candidate gene models in evaluating chemotherapy sensitivity.

### Evaluation of the prognostic value of PTX score and CBP score

We calculated the PTX score and CBP score of 320 HGSOC samples with complete survival information from TCGA-OV database using the formula in "Evaluation of the chemotherapy sensitivity of PTX score and CBP score" section, and we used “survival” package for Kaplan–Meier survival analysis. The patients were divided into two groups according to the median score. Then we evaluated the influence of score on HGSOC patients’ overall survival (OS) rate, and the criterion was same as "Survival analysis" section.

### Statistical methods

The data were analyzed statistically by R Studio (Version 3.7.0), Perl (Version 5.28.1) and GraphPad Prism (Version 8.0.2.263). Student t test and Wilcoxon signed rank test were used to analyze the differences between the two groups. And the correlation was analyzed by Spearman-rank correlation. P < 0.05 was considered to be statistically significant.

## Results

### Identification of DEGs in HGSOC by the microarray database

**Fig. 1:**
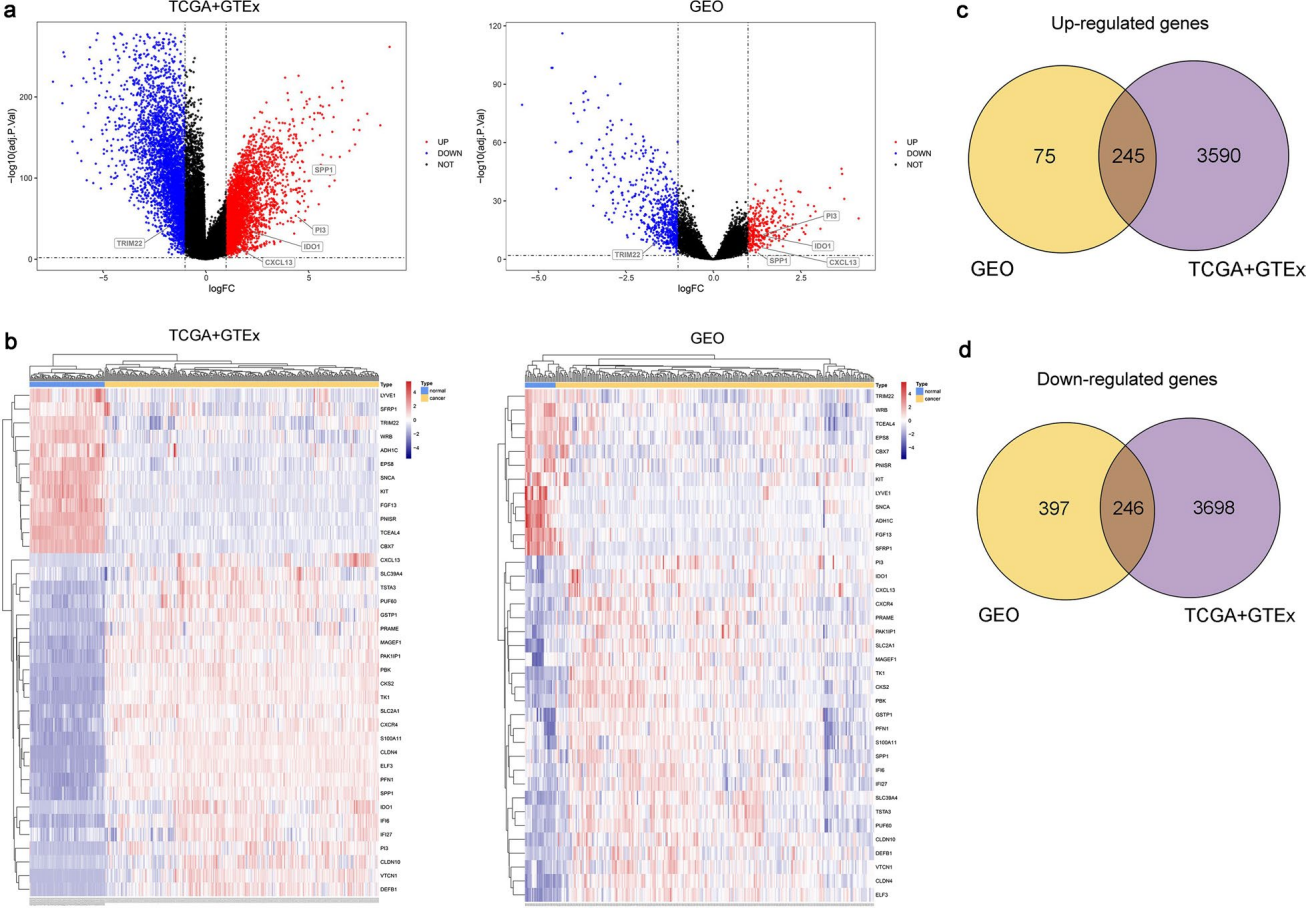
Identification of DEGs in HGSOC by the microarray database. **a** Gene expression profile volcano map. The longitudinal dotted line represents the cut-off value of ∣logFC∣ = 1, and the horizontal dotted line represents the cut-off value of adj. P = 0.05. The red colors are high expression genes of HGSOC, and the blue colors are low expression genes of HGSOC. TCGA: n = 321, GTEx: n = 88, GEO: n = 296 (cancer: 270, normal: 26). **b** Gene expression profile heat map. The red color is high expression, and the blue color is low expression. The horizontal axis is the clustering result. **c** Venn map of upregulated DEGs of HGSOC. **d** Venn map of downregulated DEGs of HGSOC

The study design was illustrated in Additional file 6: Fig.S1. According to the three gene expression profiles of GSE18520, GSE26712 and GSE40595 and two datasets of TCGA and GTEx, the chip dataset and high-throughput sequencing dataset containing both normal ovarian epithelium and HGSOC samples were respectively obtained. Volcano maps (Fig. 1a) were constructed to reflect the distribution of DEGs in GEO and TCGA + GTEx datasets, and heat maps (Fig. 1b) were constructed to indicate the expression of genes and clustering results in samples. After a series of analyses, we screened 320 and 3,835 HGSOC high expression genes and 643 and 3,944 low expression genes from the GEO and TCGA + GTEx datasets, respectively. After taking the intersection 491 genes displayed a consistent expression trend in two profiles. These genes could be subdivided into two parts: 245 (Fig. 1c) that showed high expression and 246 (Fig. 1d) that showed low expression in HGSOC tissue.

### Identification of prognosis-related DEGs and construction of PPI network and core module

**Fig. 2:**
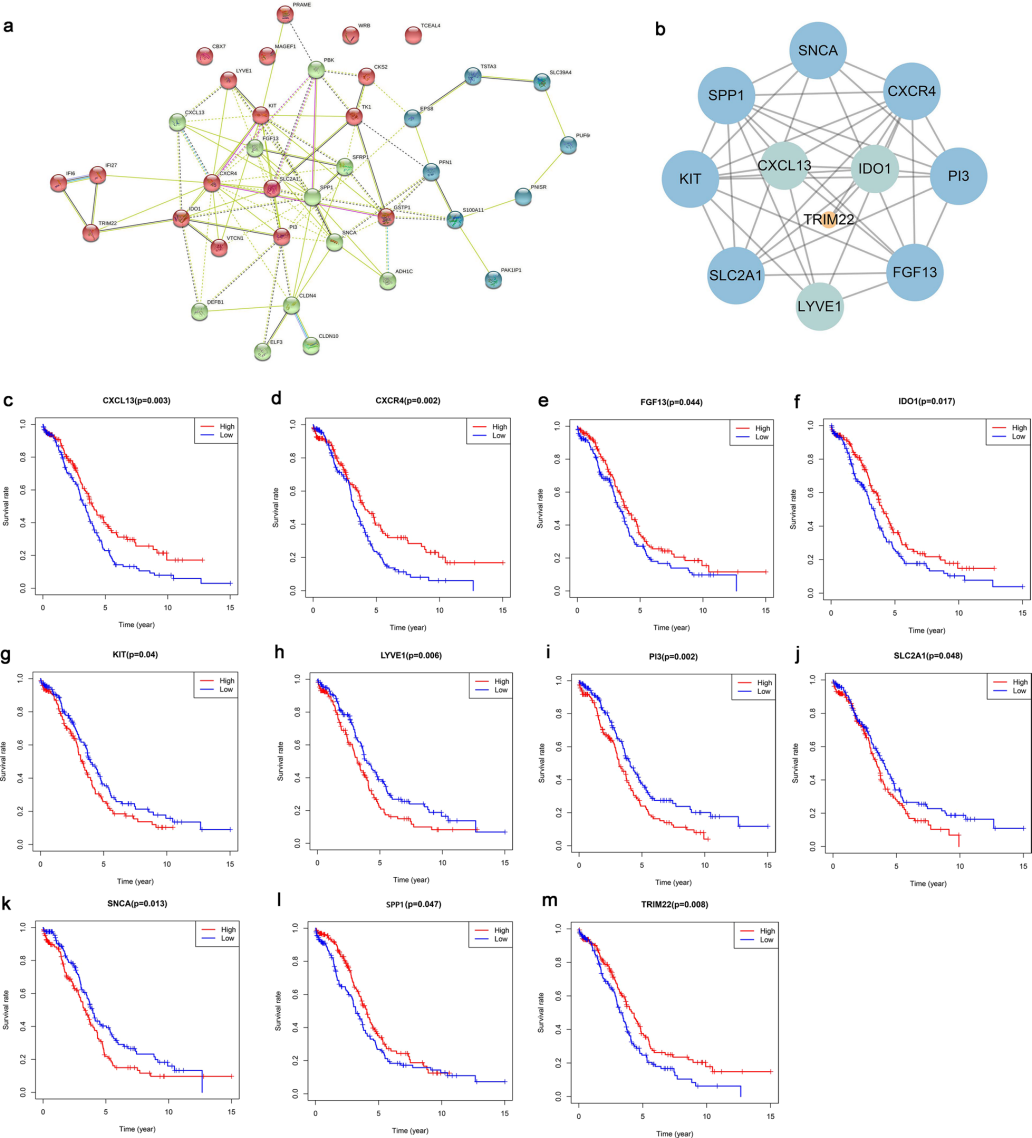
Identification of prognosis-related DEGs. **a** Prognosis-related DEGs PPI network constructed by STRING. Different colors represent the preliminary clustering by STRING according to protein functions. **b** Core module after further clustering the PPI network by MCODE. The area of the circle represents the closeness of the connection between each node and other nodes, and the more connections, the larger the area of the circle. **c**–**m**. Survival curves of hub genes by KM analysis (n = 320). The horizontal axis is time, and the longitudinal axis is the overall survival rate of corresponding time. The red color represents the group of genes with high expression, and the blue color represents the group of genes with low expression. **c** CXCL13; **d** CXCR4; **e **FGF13; **f **IDO1; **g **KIT; **h **LYVE1; **i **PI3; **j **SLC2A1; **k **SNCA; **l **SPP1; **m **TRIM22

The gene expression profiles and survival information of 320 HGSOC cases in TCGA-OV dataset were integrated according to 491 DEGs in "Identification of DEGs in HGSOC by the microarray database" section. And among them 37 genes could influence the overall survival (OS) rate by Kaplan–Meier analysis (P < 0.05) (Additional file 2: Table S2). We mapped the PPI network of 37 genes and multiple sub-networks were obtained after clustering the PPI network (Fig. 2a). We screened and visualized the core module that ranked first according to the number of nodes, the number of sides and the score value (Fig. 2b). This module included 11 proteins and had a closer interaction than others, which might influence the genesis and prognosis of HGSOC as a critical protein complex or a function module.

Eleven genes encoding core module proteins were used as hub genes, and survival curve was drawn according to the information of TCGA-OV database (Fig. 2c–m). The results showed that low expression of CXCL13, CXCR4, FGF13, IDO1, SPP1 and TRIM22 in HGSOC was associated with decreased overall survival rate of patients, and the high expression of KIT, LYVE1, PI3, SLC2A1 and SNCA had a connection with the decreased overall survival rate of patients.

### Analysis about tumor-infiltrating immunocytes and immune functional features

**Fig. 3:**
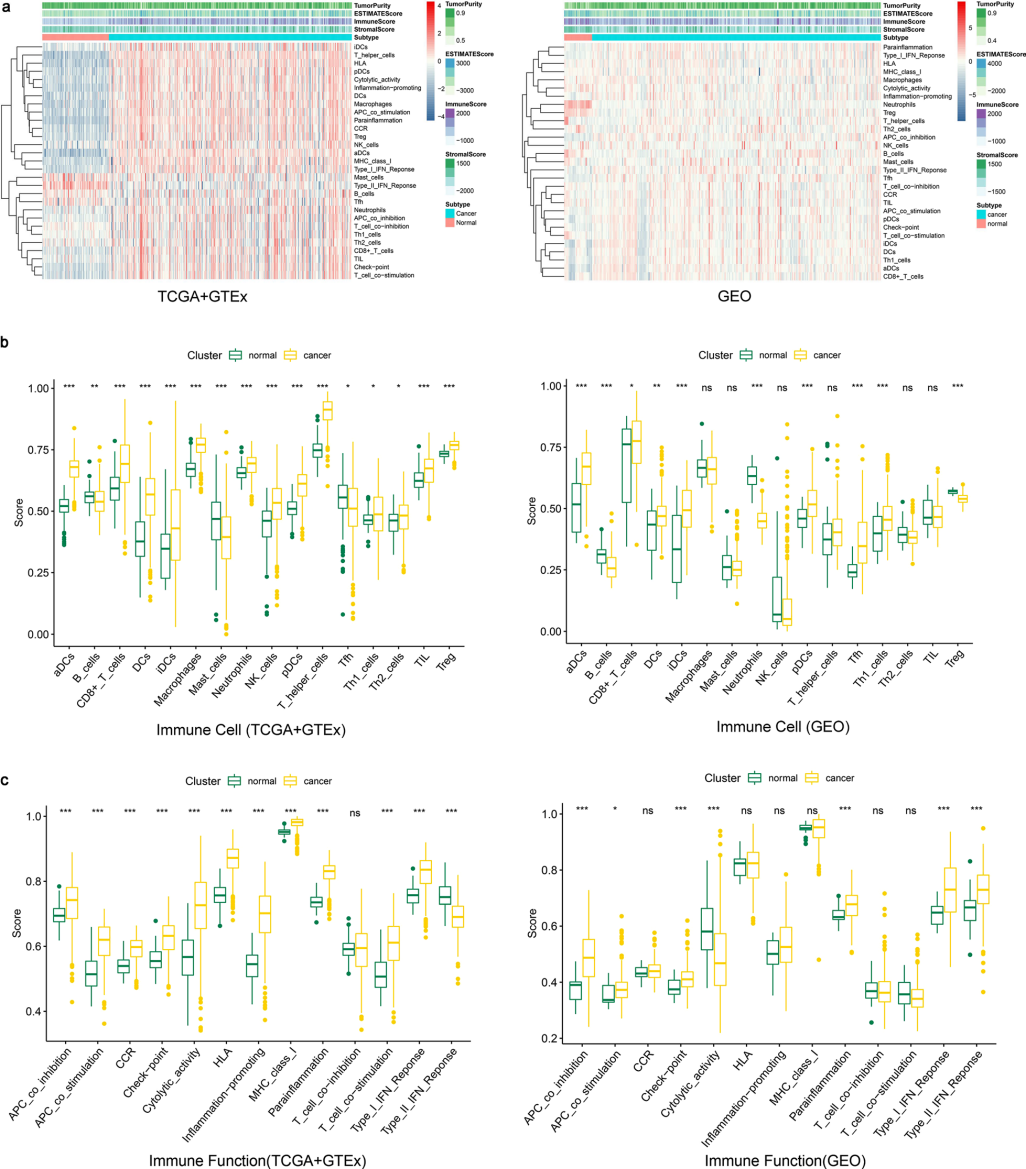
Tumor-infiltrating immunocytes and immune functional features of the cohorts. **a** Immune characteristic heat maps of TCGA + GTEx cohort(left) and GEO cohort (right). TCGA: n = 321, GTEx: n = 88, GEO: n = 296 (cancer: 270, normal: 26). Tumor purity, ESTIMATE total score, immune cell score, stroma score, immune infiltrating cells, immune function and pathway activity of every single sample are annotated by color depth. **b** Comparation of immune infiltrating cells between normal ovarian epithelium samples (green) and HGSOC samples (yellow) in TCGA + GTEx cohort (left) and GEO cohort (right). The Boxes horizontal line represents the median value, and the top line and bottom line represent the 25th and 75th percentiles (interquartile range). The whiskers encompass 1.5 times the interquartile range. The differences were analyzed by Wilcoxon signed rank test (*, P < 0.05; **, P < 0.01; ***, P < 0.0001). **c** Comparations of immune functions and pathway activities between normal ovarian epithelium samples (green) and HGSOC samples (yellow) in TCGA + GTEx cohort (left) and GEO cohort (right), and the annotation is same as **b**

We used ssGSEA to analyze and visualize the immune infiltrating cells and immune functional features from normal ovarian epithelium and HGSOC samples in TCGA + GTEx cohort(left) and GEO cohort(right) (Fig. 3a). The differences of immune cells scores, stroma scores, immune infiltrating cells, immune functions and activities of pathways between normal samples and HGSOC samples could be directly observed, and it was more obvious in TCGA + GTEx cohort.

We found that the infiltration of dendritic cells (DCs), activated dendritic cells (aDCs), immature dendritic cells (iDCs), plasmacytoid dendritic cells (pDCs), CD8 + T cells and Th1 cells generally increased while the infiltration of B cells decreased when further compared the immune infiltrating cells, immune functions and activities of pathways between normal samples and HGSOC samples in two cohorts (Fig. 3b). Similarly, the immune functions or activities of pathways including the APC co-suppression, APC co-stimulation, immune checkpoints, parainflammation and I-type IFN reaction enhanced in HGSOC samples (Fig. 3c).

Fig.S2a showed the proportion of 22 kinds of immune cells in each sample. Finally, we integrated the results of ssGSEA and CYBERSORT analysis, and found that the dendritic cells infiltration was consistently increased in HGSOC samples (Fig.S2b).

### Correlation analysis among prognosis-related hub genes and tumor-infiltrating immunocytes, as well as immune checkpoints

Previous studies confirmed that immune system played an important part in the genesis, progression, prognosis and treatment of ovarian cancer. We found that the immunocyte infiltration, immune functions and related activities of pathways of HGSOC patients greatly changed from "Analysis about tumor-infiltrating immunocytes and immune functional features" part. Thus, we took further correlation analysis among HGSOC prognosis-related genes from "Identification of prognosis-related DEGs and construction of PPI network and core module" part and tumor-infiltrating immunocytes, as well as immune checkpoints.

**Fig. 4:**
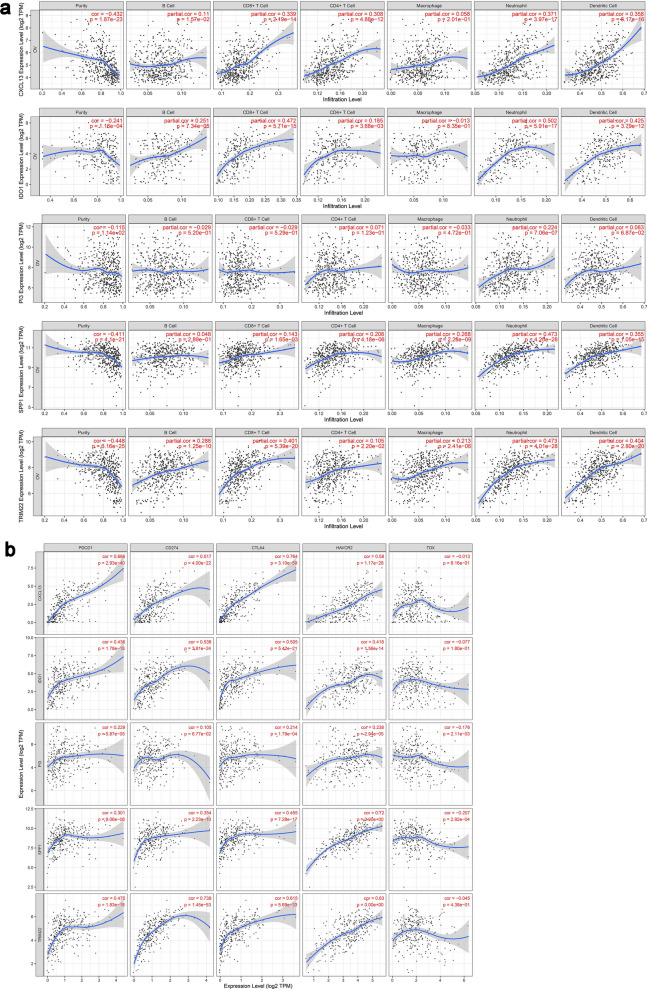
Correlation analysis among prognosis-related hub genes and immune features. **a** Visualization of correlation analysis results between five genes and immune infiltrating cells. The vertical axises are CXCL13, IDO1, PI3, SPP1 and TRIM22 from top to bottom; From left to right the horizontal axises are tumor purity, B cells, CD8 + T cells, CD4 + T cells, macrophages, neutrophils and dendritic cells. **b** Visualization of correlation analysis results between five genes and immune checkpoint gene expressions. The vertical axises are CXCL13, IDO1, PI3, SPP1 and TRIM22 from top to bottom. The horizontal axises are PDCD1, CD274, CTLA4, HAVCR2 and TOX from left to the right. TIMER website includes 303 samples from TCGA

According to the expression profile of serous OC samples from TCGA database, we analyzed the relevance between the expressions of 11 genes including CXCL13 and the contents of immune infiltrating cells like B cells, CD8 + T cells and so on from samples (Additional file 3: Table S3). Further, the analysis of the expression collinearity between 11 genes and 5 immune checkpoint genes including PDCD1, CD274, CTLA4, HAVCR2 and TOX was made (Additional file 4: Table S4). We screened CXCL13, IDO1, PI3, SPP1 and TRIM22 in all with the strongest correlation with HGSOC immune infiltrating cells and immune checkpoints according to related coefficients and P values (Tables 1, 2), and visualized the results by scatter diagram (Fig. 4a, b).

**Table 1:** The results of relevance analysis between DEPHGs of HGSOC and immunocytes (top five)

	Variable	B Cell	CD8 + T Cell	CD4 + T Cell	Macrophage	Neutrophil	Dendritic Cell
CXCL13	Partial cor	**0.110**	**0.339**	**0.308**	0.058	**0.371**	**0.358**
	P.value	0.016	0.000	0.000	0.201	0.000	0.000
IDO1	Partial cor	**0.251**	**0.472**	**0.185**	− 0.013	**0.502**	**0.425**
	P.value	0.000	0.000	0.004	0.835	0.000	0.000
PI3	Partial cor	− 0.029	− 0.029	0.071	− 0.033	**0.224**	0.083
	P.value	0.520	0.529	0.123	0.472	0.000	0.069
SPP1	Partial cor	0.048	**0.143**	**0.208**	**0.268**	**0.473**	**0.355**
	P.value	0.289	0.002	0.000	0.000	0.000	0.000
TRIM22	Partial cor	**0.288**	**0.401**	**0.105**	**0.213**	**0.473**	**0.404**
	P.value	0.000	0.000	0.022	0.000	0.000	0.000

^*^The bold fonts in Table 1 indicate significant correlations between hub genes and immune infiltrating cells (P < 0.05). The correlation degree is judged by the correlation coefficient cor. Positive cor means a positive correlation and negative cor means a negative correlation. The closer the absolute value of cor is to 1, the stronger the correlation is

**Table 2:** The results of relevance analysis between 5 gene expressions and immune checkpoint expressions

	Variable	PDCD1	CD274	CTLA4	HAVCR2	TOX
CXCL13	Cor	**0.666**	**0.517**	**0.764**	**0.580**	− 0.013
	P.value	0.000	0.000	0.000	0.000	0.816
IDO1	Cor	**0.436**	**0.538**	**0.505**	**0.418**	− 0.077
	P.value	0.000	0.000	0.000	0.000	0.180
PI3	Cor	**0.229**	0.105	**0.214**	**0.238**	− **0.176**
	P.value	0.000	0.068	0.000	0.000	0.002
SPP1	Cor	**0.301**	**0.354**	**0.455**	**0.720**	− **0.207**
	P.value	0.000	0.000	0.000	0.000	0.000
TRIM22	Cor	**0.475**	**0.739**	**0.615**	**0.630**	− 0.045
	P.value	0.000	0.000	0.000	0.000	0.438

^*^The annotation is same as Table 1

In addition to TIMER analysis, CYBERSORT was used to analyze the relationship between five genes and immune infiltrating cells in TCGA-HGSOC. The results showed that CXCL13 had a certain linear relationship with CD8 + T cells and CD4 + T cells. IDO1 and B cells, CD8 + T cells, CD4 + T cells; PI3 and neutrophils; SPP1 and CD4 + T cells, neutrophils, dendritic cells; TRIM22 and CD8 + T cells, CD4 + T cells, macrophages and other immune cells were consistent with TIMER results (Fig.S2c). It is suggested that CXCL13, IDO1, PI3, SPP1 and TRIM22 were closely related to the immune process of HGSOC.

The results indicated that CXCL13, IDO1, PI3, SPP1 and TRIM22 played significant roles in the immune process of HGSOC, which could participate in a variety of immunocytes infiltration and were closely related to the gene expressions of immune checkpoints. Especially the co-expression between CXCL13 and immune checkpoint CTLA4, the co-expression between SPP1 and immune checkpoint HAVCR2, as well as the co-expression between TRIM22 and immune checkpoint CD274 were strong (cor > 0.7) (Table 2; Fig. 4b).

### Histological verification of CXCL13, IDO1, PI3, SPP1, TRIM22

**Fig. 5:**
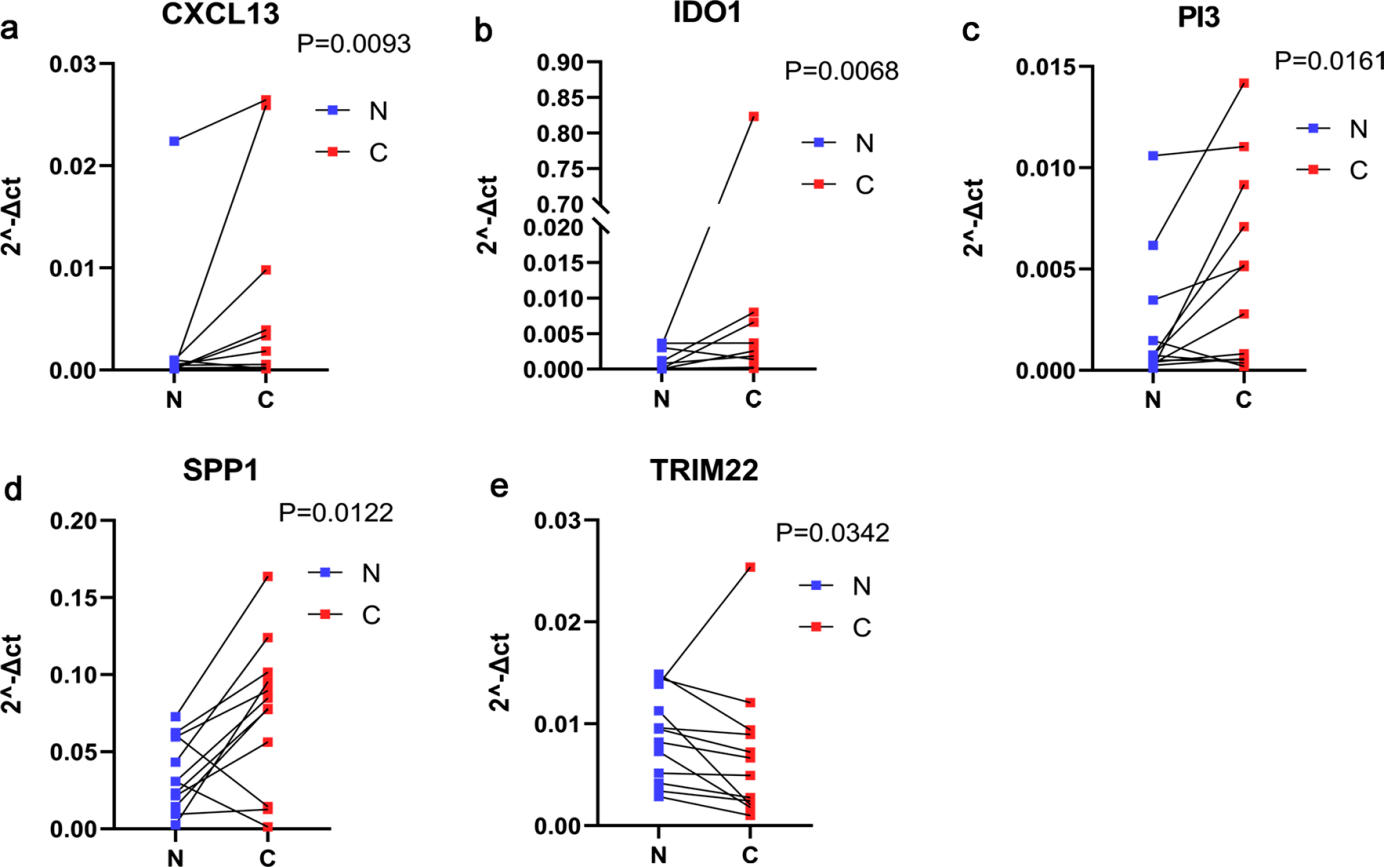
Histological verification. RT-qPCR results in the 12 pairs of HGSOC tissues and tumor-adjacent tissues showing the decreased expression of CXCL13 (**a**), IDO1 (**b**), PI3 (**c**), SPP1 (**d**), and the elevated expression of TRIM22 (**e**) in HGSOC tissues. Red points represent the HGSOC tissues and blue points represent the paired paracancerous ovarian tissues. Significance is determined by Student t test

Compared with the paracancerous tissues, the expression of CXCL13(P = 0.0093), IDO1(P = 0.0068), PI3(P = 0.0161), SPP1(P = 0.0122) in tumor tissues was significantly increased, while TRIM22(P = 0.0342) was significantly decreased. These results were consistent with the bioinformatics analysis (Fig. 5).

### GSEA analysis of CXCL13, IDO1, PI3, SPP1, TRIM22

**Fig. 6:**
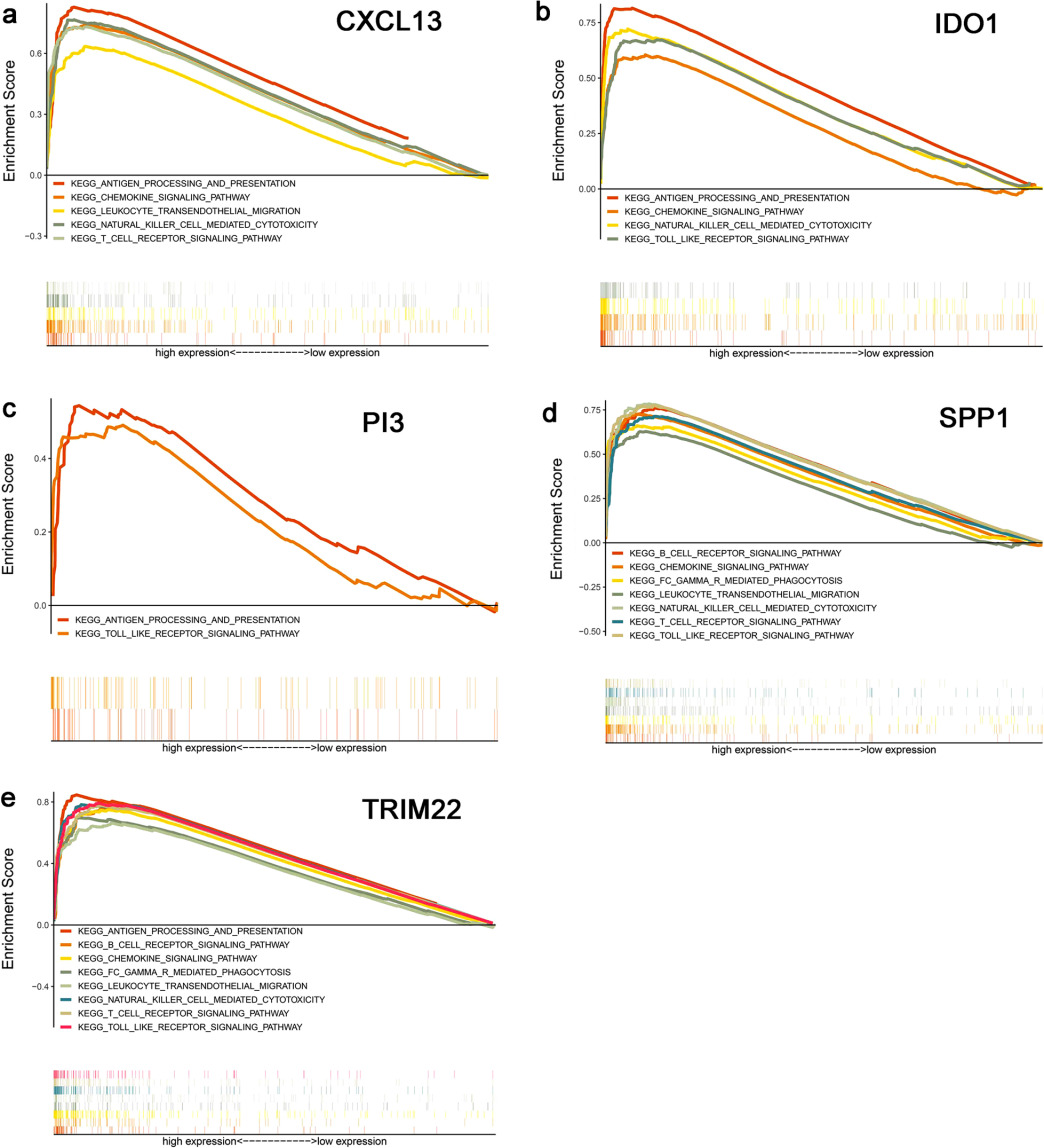
GSEA analysis. Enrichment immune-related pathways of CXCL13 (**a**), IDO1 (**b**), PI3 (**c**), SPP1 (**d**), TRIM22 (**e**) (n = 321). The vertical axis represents ES, while the horizontal axis represents the gene set distribution. The GSEA software estimates the statistical significance (nominal P value) of the ES by using an empirical phenotype-base permutation test procedure and adjust the estimated significance level of account for multiple hypothesis [43]

The results of GSEA analysis revealed that CXCL13 was enriched in the pathways of antigen processing and presentation, natural killer cell mediated cytotoxicity, chemokine signaling, et al. (Fig. 6a). IDO1 was enriched in the pathways of natural killer cell mediated cytotoxicity, antigen processing and presentation, et al. (Fig. 6b). PI3 was enriched in the pathways of antigen processing and presentation, toll like receptor signaling (Fig. 6c). SPP1 was enriched in the pathways of chemokine signaling, natural killer cell mediated cytotoxicity, et al. (Fig. 6d). TRIM22 was enriched in the pathways of chemokine signaling, T cell receptor signaling, natural killer cell mediated cytotoxicity, et al. (Fig. 6e).

### Correlation analysis of CXCL13, IDO1, PI3, SPP1, TRIM22 with clinicopathological features and prognosis

According to the clinical information of patients in TCGA-OV database, the correlation analysis between the expression of five genes and the clinicopathological features of HGSOC showed that the expression of residual genes had no significant correlation with clinical stage and grade status, except that the patients with high expression of SPP1 were more prone to lymphatic invasion (Additional file 8: Fig.S3a–c).

Consistent with the results of KM analysis in "Identification of prognosis-related DEGs and construction of PPI network and core module" section, COX regression analysis suggested that CXCL13, IDO1, SPP1, TRIM22 were protective factors for the prognosis of HGSOC patients, while PI3 was a risk factor (Additional file 8: Fig.S3d).

### Analysis based on chemotherapy sensitivity of tumor immune-related genes

We divided patients from GSE15622 dataset into paclitaxel resistant group (PR), paclitaxel sensitive group (PS), carboplatin resistant group (CR) and carboplatin sensitive group (CS) according to their information about chemotherapy sensitivity, The results of GO analysis showed that there were some degrees of immune activation both in two chemosensitive groups. For example, the processes of leukocyte migration, regulation of leukocyte migration were enriched in PS group (Additional file 9: Fig.S4a), while the processes of neutrophil degranulation, neutrophil activation, neutrophil mediated immunity were enriched in CS group (Additional file 9: Fig.S4c).

**Fig. 7:**
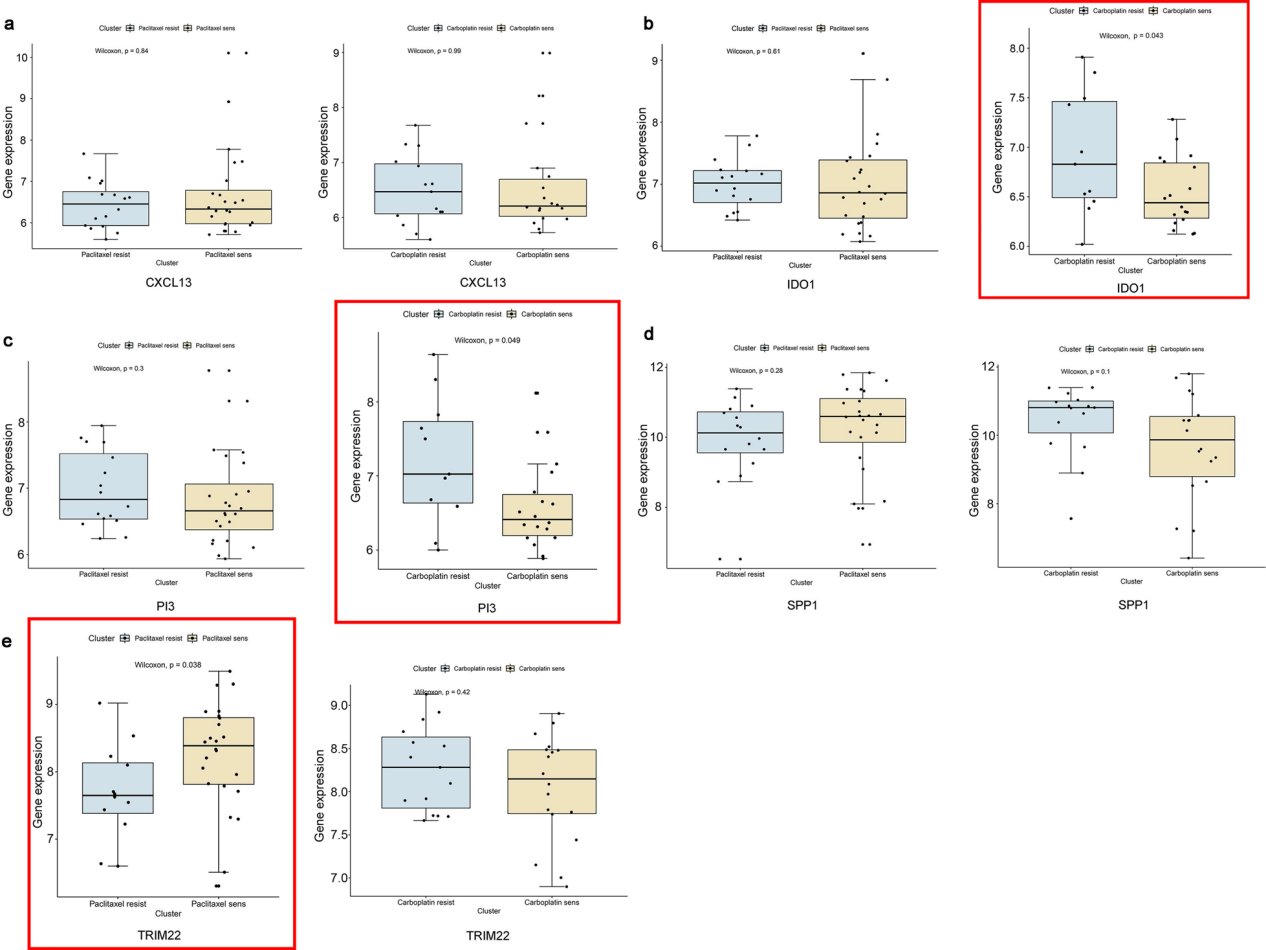
Correlation analysis between tumor immune-related genes and chemotherapy sensitivity. Comparation of single gene expression between paclitaxel resistant group (n = 12) and paclitaxel sensitive group (n = 24) in ovarian cancer patients (left), and the comparation of single gene expression between carboplatin resistant group (n = 11) and carboplatin sensitive group (n = 18) (right). **a** CXCL13; **b** IDO1; **c** PI3; **d** SPP1; **e** TRIM22

Furthermore, we compared the expressions of CXCL13, IDO1, PI3, SPP1 and TRIM22 between groups. The results showed that TRIM22 was significantly different between PR and PS group (Fig. 7e), while the expressions of IDO1 and PI3 were significantly different between CR and CS group (Fig. 7b, c). And it indicated that single gene expression of IDO1, PI3 and TRIM22 could influence the chemotherapy sensitivity of ovarian cancer patients. Patients with high expression of TRIM22 might be more sensitive to paclitaxel, while patients with low expression of IDO1 and PI3 might be more sensitive to carboplatin.

### Application evaluation of prediction models PTX score and CBP score

We found that single gene expressions of IDO1, PI3 and TRIM22 could influence the chemotherapy sensitivity of OC patients in "Analysis based on chemotherapy sensitivity of tumor immune-related genes" section. At the same time, the previous analysis of the study showed that the coding proteins of CXCL13, IDO1, PI3, SPP1 and TRIM22 had a strong potential relevance. And they were all closely related to the immunocyte infiltration and immune checkpoints of HGSOC. Thus, the scoring model based on the gene expressions of CXCL13, IDO1, PI3, SPP1 and TRIM22 from GSE15622 dataset and constructed by lasso algorithm could better predict the sensitivity of ovarian cancer patients to chemotherapeutic drugs than which based on single gene.

The construction of PTX score prediction model according to gene expression profiles of patients with paclitaxel treatment from GSE15622 was as follows:

$$PTX score = - 0.41 \times IDO1 - 0.57 \times PI3 + 0.18 \times SPP1 + 0.83 \times TRIM22$$

**Fig. 8:**
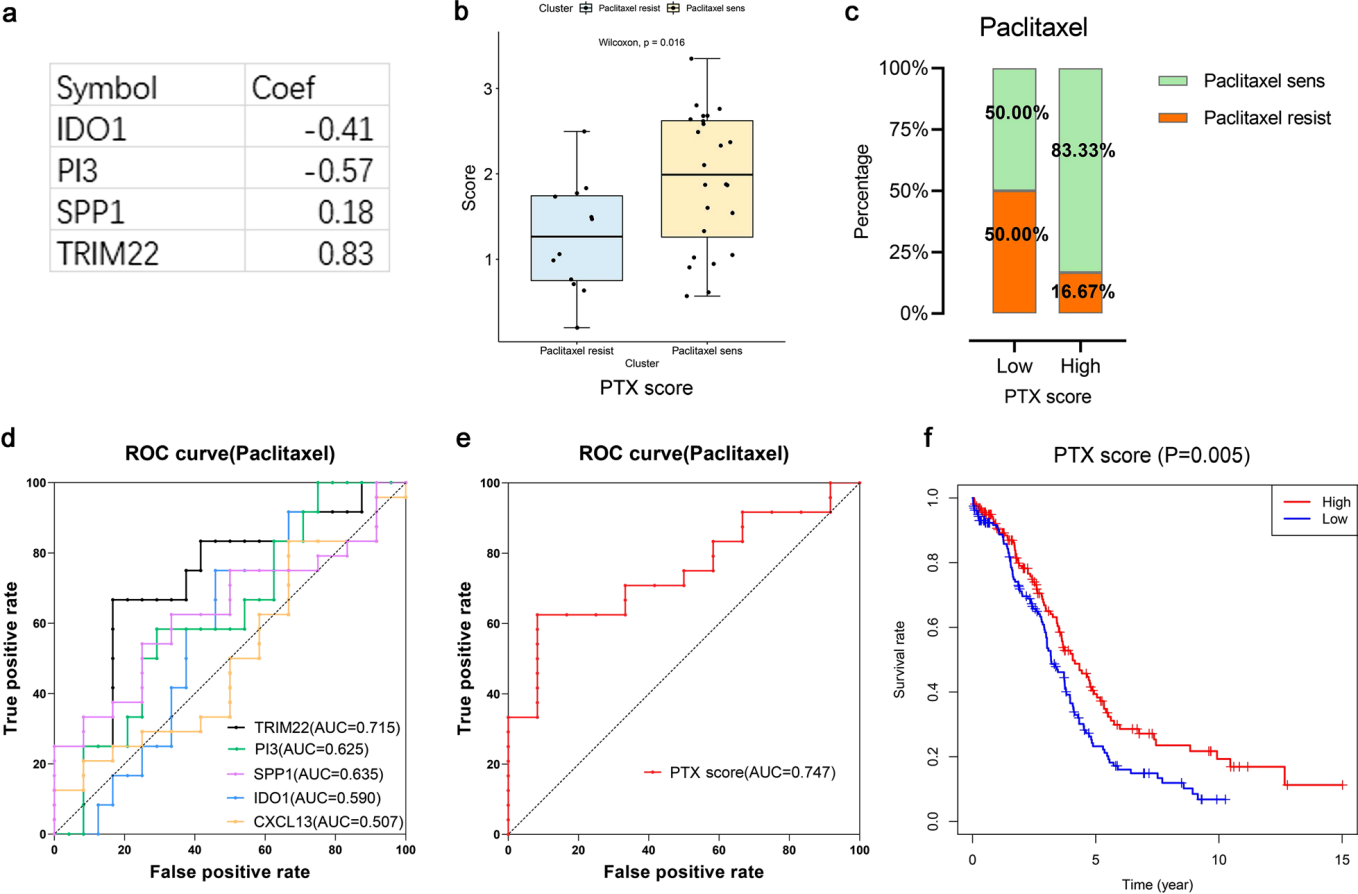
PTX score paclitaxel sensitivity prediction model. **a** Gene coefficients of PTX score paclitaxel sensitivity prediction model. **b** Comparation of PTX scores between paclitaxel resistant group (blue) and paclitaxel sensitive group (yellow). **c** Proportions of paclitaxel resistant (orange) and sensitive (green) patients in high PTX score group and low PTX score group. **d** ROC curves of paclitaxel sensitivity in ovarian cancer patients evaluated by TRIM22 (black), PI3 (green), SPP1 (purple), IDO1 (blue) and CXCL13 (yellow). The larger the AUC, the more accurate the judgment. **e** ROC curve of paclitaxel sensitive in ovarian cancer patients evaluated by PTX score (red). **f** Survival curves by KM analysis of PTX score in TCGA database (n = 320). The horizontal axis is the time, and the longitudinal axis is the overall survival rate of corresponding time. The red color represents high PTX score group and blue color represents low PTX score group

We calculated PTX scores in every sample and found it had a significant difference between PR group and PS group. The PTX scores were generally higher in PS group (Fig. 8b). Besides, the percentage of patients who were sensitive to paclitaxel treatment in high-PTX score group was greatly increased when patients were divided into high-PTX score group and low-PTX score group according to the median value (Fig. 8c). The AUC of ROC curve showed that the accuracy of judging sensitivity of patients to paclitaxel by PTX score (AUC = 0.747) was higher than those by the expression of single gene including TRIM22 (AUC = 0.715), PI3 (AUC = 0.625), SPP1 (AUC = 0.635), IDO1 (AUC = 0.590) and CXCL13 (AUC = 0.507) (Fig. 8d, e). Then we calculated PTX scores of 320 HGSOC cases in TCGA-OV database according to the model and found that the overall survival rate of high-PTX score group increased significantly (Fig. 8f).

Next, the construction of CBP score prediction model according to gene expression profiles of patients with carboplatin treatment from GSE15622 was as follows:

$$CBP score = 1.01 \times CXCL13 - 1.82 \times IDO1 - 1.10 \times PI3 - 0.14 \times SPP1 - 1.18 \times TRIM22$$

**Fig. 9:**
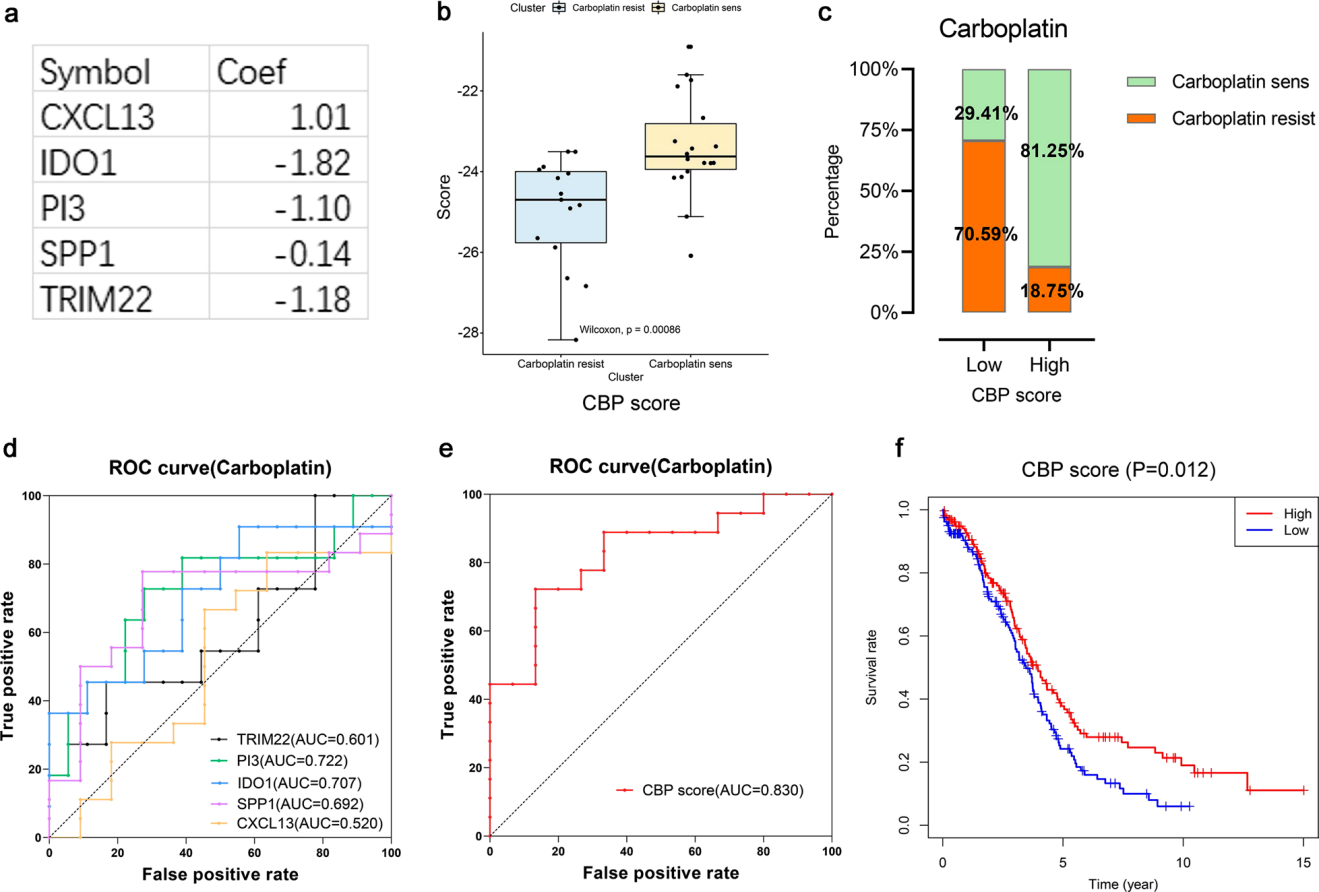
CBP score carboplatin sensitivity prediction model. **a** Gene coefficients of CBP score carboplatin sensitivity prediction model. **b** Comparation of CBP scores between carboplatin resistant group (blue) and carboplatin sensitive group (yellow). **c** Proportions of carboplatin resistant (orange) and sensitive (green) patients in high CBP score group and low CBP score group. **d** ROC curves of carboplatin sensitivity in ovarian cancer patients evaluated by TRIM22 (black), PI3 (green), SPP1 (purple), IDO1 (blue) and CXCL13 (yellow). The larger the AUC, the more accurate the judgment. **e** ROC curve of carboplatin sensitive in ovarian cancer patients evaluated by CBP score (red). **f** Survival curves by KM analysis of CBP score in TCGA database (n = 320). The horizontal axis is the time, and the longitudinal axis is the overall survival rate of corresponding time. The red color represents high CBP score group and blue color represents low CBP score group

We calculated CBP scores in every sample and found it had a significant difference between CR and CS group. The CBP scores were generally higher in CS group (Fig. 9b). Besides, the percentage of patients who were sensitive to carboplatin treatment in high-CBP score group was greatly increased when patients were divided into high-CBP score group and low-CBP score group according to the median value (Fig. 9c). The AUC of ROC curve showed that the accuracy of judging sensitivity of patients to carboplatin by CBP score (AUC = 0.830) was higher than those by the expression of single gene including TRIM22 (AUC = 0.601), PI3 (AUC = 0.722), SPP1 (AUC = 0.707), IDO1 (AUC = 0.692) and CXCL13 (AUC = 0.520) (Fig. 9d, e). Then we calculated CBP scores of 320 HGSOC cases in TCGA-OV database according to the model and found that the overall survival rate of high-CBP score group increased significantly (Fig. 9f).

Univariate and multivariate COX regression analysis showed that age, residual tumor lesion, CBP score and PTX score were independent prognostic factors for HGSOC patients (Additional file 9: Fig.S4e). Patients older than 70 years, HGSOC residual lesions larger than 1 cm, low CBP score, and low PTX score were more likely to have poor prognosis (Additional file 9: Fig.S4f).

The above results confirmed that two models based on the gene expressions of CXCL13, IDO1, PI3, SPP1 and TRIM22 could be applied to predict the sensitivity of HGSOC patients to paclitaxel and carboplatin, and they had great judgment value. Meanwhile, the overall survival rate of patients from high chemotherapy sensitivity group was higher, which indicated that PTX score and CBP score could be well used to predict the chemotherapy sensitivity and prognosis risk of ovarian cancer patients.

## Discussion

Ovarian cancer is a common gynecological tumor along with poor prognosis, the outcome of which is associated with immunocyte infiltration. Difficulty in early diagnosis and the recurrence because of resistance to chemotherapy generally makes the high mortality of ovarian cancer patients. HGSOC is usually treated with surgical resection combined with chemotherapy of paclitaxel and carboplatin (bevacizumab is added in some cases) as the most common subtype of epithelial ovarian cancer [12]. HGSOC often happens to elderly women in advanced stage of FIGO, and they intend to develop drug resistance with time passing by, which leads to a bad outcome although chemotherapeutic drugs work well in the initial stage.

The poor prognosis of HGSOC is correlated with immunocyte infiltration and immunotherapy, but the research about comprehensive analysis of these three aspects is rare. Our study screened HGSOC prognosis-related genes and explored whether they were equally important in tumor immune response and treatment. Finally, we got prognostic markers with potential application value and could also be used as immunotherapy molecular targets of HGSOC. At the same time, score models based on these target genes could effectively assess the chemosensitivity and prognosis of patients.

First, we got 491 DEGs of HGSOC from GEO, TCGA and GTEx databases, and after prognosis analysis we found 37 genes could influence overall survival rate of patients. And we screened one core module which included 11 hub genes by PPI to get potential molecular complex. What’s more, the analysis about immune characteristics of HGSOC samples showed that immune infiltrating cells, immune functions and activities of pathways changed greatly compared to normal ovarian epithelium, especially the increase of immune infiltration of dendritic cells and the enhanced activity of APC function and immune checkpoints.

Immunocytes play an essential role in tumor tissue, and an increasing number of studies has shown that they also have clinicopathological significances in predicting prognosis and efficacy [46, 47]. Immune checkpoint inhibitors (ICPIs) can prevent immune escape of tumor and reactivate the immune system to produce an anti-tumor response by destroying important targets in the process of tumor immune tolerance. The past studies suggested that samples of tumor patients who were reactive to PD1 immune checkpoint inhibitors usually had higher densities of CD3 + T cells, CD8 + T cells and PD-1 + T cells in both invasive margin and center of tumor [48]. Some researchers also found that high level infiltration of CD8 + TILs in HGSOC stroma was associated with higher overall survival rate [49]. Thus, we took further immune evaluation about immune checkpoints and immune infiltrating cells according to 11 hub genes. The results showed that CXCL13, IDO1, PI3, SPP1 and TRIM22 could participate in a variety of immunocytes infiltration and were closely related to the gene expressions of immune checkpoints.

In particular, CXCL13 and immune checkpoint CTLA, SPP1 and immune checkpoint HAVCR2, as well as TRIM22 and immune checkpoint CD274 showed strong collinear expression. It also indicated that these five genes played significant roles in the immune process of HGSOC as potential immunotherapy molecular targets. What’s more, the results of RT-qPCR verified the results of DEGs analysis of public databases. At the same time, single-gene GSEA results showed that the five genes were closely related the immune-related pathways of antigen processing and presentation, T cell receptor signaling and so on.

Lo et al. have reported that the densities of CD3 + T cells, CD8 + T cells and PD-1 + T cells increased in HGSOC after chemotherapy of platinum, and the increase of these T cell subtypes was associated with the presence of immunocytes before treatment, which suggested that platinum chemotherapy could induce expected immune reaction if the necessary immunocytes had already been in the tumor [49]. These studies indicated that the chemosensitivity of HGSOC patients was closely related to the cancer immune microenvironment. According to the dataset expression profiles from GSE15622 and information of patients’ sensitivity to chemotherapeutic drugs, our analysis found that patients with high expression of TRIM22 were more sensitive to paclitaxel, while patients with low expression of IDO1 and PI3 were more sensitive to carboplatin.

At present, the roles of CXCL13, IDO1 and SPP1 in immune process of tumor have been studied. As a chemokine, CXCL13 binds to its homologous receptor CXCR5, participates in the migration and recruitment of lymphocytes, which helps to enhance the immune response of tumor host [50, 51]. CXCL13 could enhance the effectiveness of PD-1 blocking therapy in ovarian cancer [52]. IDO1 can induce the production of immunosuppressive molecule Tregs by inhibiting the function of T cells, and then produce a series of immunosuppressive effects [53, 54]. Now a variety of small molecule inhibitors targeting IDO1 have entered the clinical research stage [55]. SPP1, which is highly expressed in non-small cell lung cancer, breast cancer and colorectal cancer, evades tumor immunization by regulating the polarization of macrophages in tumor microenvironment, recruiting and inhibiting the activation of T cells, and is related to the prognosis of patients and resistance of drugs [56–62]. Up-regulation of SPP1 was detected in plasma of patients with OC [63]. And in patients with recurrent ovarian cancer, the expression of SPP1 increased in the early stage, which can detect cancer recurrence earlier than that of CA125 alone [64].

PI3 (code Elafin) has been confirmed that it’s highly expressed in HGSOC and is associated with low overall survival rate [65]. PI3 can reduce the sensitivity of epithelial ovarian cancer (EOC) cells to cisplatin and other drugs, but its correlation with immune function has not been revealed [66]. TRIM22 could be greatly upregulated under the stimulations of IFN, LPS and p53 [67]. Current research shows that TRIM22 is highly expressed in glioma and can promote the proliferation of tumor cells, while it plays an anti-cancer role in endometrial cancer [68, 69]. The function and mechanism of TRIM22 in tumor progression need to be further studied.

Past studies showed that CXCL13, IDO1, PI3, SPP1 and TRIM22 were closely correlated with the prognosis, immunization and chemotherapy sensitivity of tumor. We constructed PTX score model to predict the sensitivity of paclitaxel and CBP score model to predict the sensitivity of carboplatin based on the gene expressions of CXCL13, IDO1, PI3, SPP1 and TRIM22. The results showed that they could better predict the sensitivity of ovarian cancer patients to chemotherapeutic drugs than which based on single gene like IDO1, PI3 and TRIM22. After TCGA database was incorporated, we found that the overall survival rate of patients from high chemotherapy sensitivity group (the score was higher than the median) was higher, and CBP score and PTX score could act as independent prognostic factors together with age and tumor residual lesions, which indicated that PTX score and CBP score could be well used to predict the chemotherapy sensitivity and prognosis of ovarian cancer patients.

## Conclusions

In conclusion, through comprehensive bioinformatics analysis, 5 candidate genes—CXCL13, IDO1, PI3, SPP1 and TRIM22— were identified, which were closely related to the prognosis, immunocyte infiltration, immune checkpoints, and chemotherapy sensitivity of HGSOC. And based on this, two scoring models—PTX score and CBP score— were constructed to effectively predict chemotherapy sensitivity to paclitaxel and carboplatin and the prognosis for patients with HGSOC. Our exploratory study may provide potential biomarkers and molecular targets for chemotherapy for HGSOC, so as to help improve clinical outcomes of patients.

## Supplementary Information

**Additional file 1: Table S1**. 29 immune signatures represented as 29 different gene sets.

**Additional file 2: Table S2**. The results of KM analysis of DEGs of HGSOC based on TCGA database.

**Additional file 3: Table S3**. The results of relevance analysis between DEPHGs of HGSOC and immunocytes (all results).

**Additional file 4: Table S4**. The results of relevance analysis between gene expressions and immune checkpoint expressions (all results).

**Additional file 5: Table S5**. Primer Sequences in RT-qPCR.

**Additional file 6: Fig. S1**. A flow chart showing the whole procedures in this study.

**Additional file 7: Fig. S2**. CYBERSORT analysis showing the immune characteristics in HGSOC. a. The proportion of 22 kinds of immune cells in each sample. b. Comparation of immune infiltrating cells between normal ovarian epithelium samples (green) and HGSOC samples (yellow) in TCGA+GTEx cohort (tumor tissues form TCGA: n=321, normal tissues from GTEx: n=88). The differences were analyzed by Wilcoxon signed rank test. c. Association between 22 kinds of immune cells and 5 genes expression. Red indicates negative correlation whereas blue indicates positive correlation. Correlation coefficient are labeled on the junction points.

**Additional file 8: Fig. S3**. Clinicopathological features and prognosis correlation analysis. a-c. Association between genes expression and clinicopathological features in HGSOC. Significance is determined by Wilcoxon rank sum test (*, P<0.05). a. Clinical stage status (I: 1 case; II: 23 cases; III: 295 cases; IV: 57 cases). b. Clinical grade status (G1: 1 case; G2: 45 cases; G3: 322 cases; G4: 1 case). c. Lymphatic invasion (NO: 48 cases; YES: 101 cases). d. COX regression analysis of CXCL13, IDO1, PI3, SPP1 and TRIM22 (n=320). The points represent the HRs, the horizontal line length represents the 95% CI of each group, and the vertical dashed line represents HR=1.0. HR>1 represents a risk factor whereas HR<1 represents a protective factor.

**Additional file 9: Fig. S4**. GO analysis of GSE15622 and COX regression analysis of TCGA-HGSOC. a-d. GO term enrichment for biological processes (BP), cellular components (CC) and molecular function (MF): a. Paclitaxel sensitive group (n=24). b. Paclitaxel resistant group (n=12). c. Carboplatin sensitive group (n=18). d. Carboplatin resistant group (n=11). e-f. Prognostic value of CBP/PTX score in HGSOC samples from TCGA-OV database (n=287): e. Univariate COX regression analysis showing the prognostic value of age (high group (n=68) ≥ 70 years), stage (high group (n=277) ≥ Stage III), tumor residual lesions (high group (n=81) ≥ 10mm) and CBP/PTX score (high group (n=144) ≥ median value). The points represent the HRs, the horizontal line length represents the 95% CI of each group, and the vertical dashed line represents HR=1.0. f. Multivariate COX regression analysis showing the prognostic value of age, stage, tumor residual lesions and CBP/PTX score.

## Data Availability

The datasets generated and analyzed during the current study are available in the GEO (https://www.ncbi.nlm.nih.gov/geo/), UCSC (http://xenabrowser.net/hub/) repository.

## Abbreviations

OC: Ovarian cancer
HGSOC: High grade serous ovarian cancer
GEO: Gene Expression Omnibus
DEGs: Differential expressed genes
DEPGs: Prognosis related DEGs
DEPHGs: Hub genes of DEPGs
IDEPHGs: Immune-related DEPHGs
OS: Overall survival
PPI: Protein–protein interaction
STRING: Search tool for the retrieval of interacting genes
MCODE: Molecular complex detection
ssGSEA: Single sample gene set enrichment analysis
TILs: Tumor-infiltrating lymphocytes
DCs: Dendritic cells
aDCs: Activated dendritic cells
iDCs: Immature dendritic cells
pDCs: Plasmacytoid dendritic cells
APC: Antigen presentation response
ICPIs: Immune checkpoint inhibitors
PTX: Paclitaxel
CBP: Carboplatin
AUC: Area under curve
