# Supplementary material for: Model constructions of chemosensitivity and prognosis of high grade serous ovarian cancer based on evaluation of immune microenvironment and immune response
Source: Cancer Cell Int. 2021 Nov 4;21:593. doi: 10.1186/s12935-021-02295-y (PMC8567582; doi:10.1186/s12935-021-02295-y)
Supplement: Supplementary file 2 — Additional file 2: Table S2. The results of KM analysis of DEGs of HGSOC based on TCGA database. [file 12935_2021_2295_MOESM2_ESM.docx]

**Supplementary Table 2** The results of KM analysis of DEGs of HGSOC based on TCGA database

| Gene symbol | Median OS in high group (years) | Median OS in low group (years) | P. value |
| --- | --- | --- | --- |
| ADH1C | 3.26 | 3.96 | 0.0050 |
| CBX7 | 3.50 | 3.72 | 0.0107 |
| CKS2 | 3.72 | 3.46 | 0.0203 |
| CLDN10 | 4.09 | 3.17 | 0.0327 |
| CLDN4 | 3.45 | 3.96 | 0.0031 |
| CXCL13 | 3.96 | 3.42 | 0.0031 |
| CXCR4 | 4.07 | 3.26 | 0.0017 |
| DEFB1 | 3.96 | 3.08 | 0.0176 |
| ELF3 | 3.61 | 4.07 | 0.0223 |
| EPS8 | 3.42 | 3.95 | 0.0067 |
| FGF13 | 3.96 | 3.42 | 0.0438 |
| GSTP1 | 3.81 | 3.42 | 0.0101 |
| IDO1 | 4.07 | 3.32 | 0.0174 |
| IFI27 | 3.81 | 3.42 | 0.0258 |
| IFI6 | 3.98 | 3.32 | 0.0415 |
| KIT | 3.26 | 3.96 | 0.0398 |
| LYVE1 | 3.18 | 3.95 | 0.0056 |
| MAGEF1 | 3.95 | 3.42 | 0.0190 |
| PAK1IP1 | 3.75 | 3.46 | 0.0200 |
| PBK | 3.98 | 3.42 | 0.0236 |
| PFN1 | 4.07 | 3.16 | 0.0005 |
| PI3 | 3.17 | 4.07 | 0.0024 |
| PNISR | 4.51 | 3.19 | 0.0060 |
| PRAME | 3.96 | 3.19 | 0.0238 |
| PUF60 | 4.07 | 3.19 | 0.0467 |
| S100A11 | 3.81 | 3.17 | 0.0314 |
| SFRP1 | 3.17 | 4.14 | 0.0031 |
| SLC2A1 | 3.42 | 3.96 | 0.0481 |
| SLC39A4 | 3.96 | 3.20 | 0.0295 |
| SNCA | 3.32 | 3.95 | 0.0128 |
| SPP1 | 3.50 | 3.67 | 0.0470 |
| TCEAL4 | 3.81 | 3.26 | 0.0483 |
| TK1 | 3.74 | 3.50 | 0.0307 |
| TRIM22 | 4.15 | 3.20 | 0.0080 |
| TSTA3 | 3.96 | 3.17 | 0.0074 |
| VTCN1 | 3.96 | 3.17 | 0.0445 |
| WRB | 4.07 | 3.42 | 0.0371 |
