# Supplementary material for: Model constructions of chemosensitivity and prognosis of high grade serous ovarian cancer based on evaluation of immune microenvironment and immune response
Source: Cancer Cell Int. 2021 Nov 4;21:593. doi: 10.1186/s12935-021-02295-y (PMC8567582; doi:10.1186/s12935-021-02295-y)
Supplement: Supplementary file 3 — Additional file 3: Table S3. The results of relevance analysis between DEPHGs of HGSOC and immunocytes (all results). [file 12935_2021_2295_MOESM3_ESM.docx]

**Supplementary Table 3** The results of relevance analysis between DEPHGs of HGSOC and immunocytes (all results)

|  | Variable | Purity | B Cell | CD8+ T Cell | CD4+ T Cell | Macrophage | Neutrophil | Dendritic Cell |
| --- | --- | --- | --- | --- | --- | --- | --- | --- |
| **CXCL13** | Partial cor | -0.432 | **0.110** | **0.339** | **0.308** | 0.058 | **0.371** | **0.358** |
|  | P.value | 0.000 | 0.016 | 0.000 | 0.000 | 0.201 | 0.000 | 0.000 |
| CXCR4 | Partial cor | -0.158 | -0.019 | 0.015 | 0.140 | -0.050 | 0.093 | 0.117 |
|  | P.value | 0.000 | 0.677 | 0.751 | 0.002 | 0.271 | 0.043 | 0.010 |
| FGF13 | Partial cor | 0.092 | -0.079 | 0.006 | -0.029 | -0.060 | -0.040 | -0.052 |
|  | P.value | 0.044 | 0.083 | 0.891 | 0.521 | 0.190 | 0.386 | 0.253 |
| **IDO1** | Partial cor | -0.241 | **0.251** | **0.472** | **0.185** | -0.013 | **0.502** | **0.425** |
|  | P.value | 0.000 | 0.000 | 0.000 | 0.004 | 0.835 | 0.000 | 0.000 |
| KIT | Partial cor | -0.101 | -0.137 | -0.119 | -0.013 | 0.129 | -0.096 | -0.091 |
|  | P.value | 0.026 | 0.003 | 0.009 | 0.776 | 0.005 | 0.036 | 0.047 |
| LYVE1 | Partial cor | -0.069 | -0.142 | -0.030 | 0.013 | 0.186 | 0.110 | 0.004 |
|  | P.value | 0.131 | 0.002 | 0.507 | 0.770 | 0.000 | 0.016 | 0.930 |
| **PI3** | Partial cor | -0.115 | -0.029 | -0.029 | 0.071 | -0.033 | **0.224** | 0.083 |
|  | P.value | 0.011 | 0.520 | 0.529 | 0.123 | 0.472 | 0.000 | 0.069 |
| SLC2A1 | Partial cor | -0.005 | -0.167 | -0.159 | -0.006 | -0.094 | 0.025 | -0.002 |
|  | P.value | 0.914 | 0.000 | 0.000 | 0.892 | 0.039 | 0.587 | 0.965 |
| SNCA | Partial cor | 0.069 | -0.127 | 0.040 | 0.025 | 0.099 | 0.026 | 0.035 |
|  | P.value | 0.131 | 0.005 | 0.383 | 0.586 | 0.030 | 0.572 | 0.447 |
| **SPP1** | Partial cor | -0.411 | 0.048 | **0.143** | **0.208** | **0.268** | **0.473** | **0.355** |
|  | P.value | 0.000 | 0.289 | 0.002 | 0.000 | 0.000 | 0.000 | 0.000 |
| **TRIM22** | Partial cor | -0.448 | **0.288** | **0.401** | **0.105** | **0.213** | **0.473** | **0.404** |
|  | P.value | 0.000 | 0.000 | 0.000 | 0.022 | 0.000 | 0.000 | 0.000 |
