# Supplementary material for: Model constructions of chemosensitivity and prognosis of high grade serous ovarian cancer based on evaluation of immune microenvironment and immune response
Source: Cancer Cell Int. 2021 Nov 4;21:593. doi: 10.1186/s12935-021-02295-y (PMC8567582; doi:10.1186/s12935-021-02295-y)
Supplement: Supplementary file 4 — Additional file 4: Table S4. The results of relevance analysis between gene expressions and immune checkpoint expressions (all results). [file 12935_2021_2295_MOESM4_ESM.docx]

**Supplementary Table 4** The results of relevance analysis between gene expressions and immune checkpoint expressions (all results)

|  | Variable | PDCD1 | CD274 | CTLA4 | HAVCR2 | TOX |
| --- | --- | --- | --- | --- | --- | --- |
| CXCL13 | Cor | **0.666** | **0.517** | **0.764** | **0.580** | -0.013 |
|  | P.value | 0.000 | 0.000 | 0.000 | 0.000 | 0.816 |
| CXCR4 | Cor | -0.019 | **0.155** | **0.133** | **0.128** | 0.040 |
|  | P.value | 0.744 | 0.007 | 0.021 | 0.026 | 0.493 |
| FGF13 | Cor | -0.042 | **-0.126** | -0.109 | **-0.197** | **0.141** |
|  | P.value | 0.463 | 0.028 | 0.058 | 0.001 | 0.014 |
| IDO1 | Cor | **0.436** | **0.538** | **0.505** | **0.418** | -0.077 |
|  | P.value | 0.000 | 0.000 | 0.000 | 0.000 | 0.180 |
| KIT | Cor | -0.058 | 0.004 | -0.035 | -0.029 | 0.105 |
|  | P.value | 0.313 | 0.939 | 0.544 | 0.615 | 0.068 |
| LYVE1 | Cor | 0.015 | 0.042 | -0.062 | **0.139** | 0.018 |
|  | P.value | 0.798 | 0.462 | 0.282 | 0.015 | 0.752 |
| PI3 | Cor | **0.229** | 0.105 | **0.214** | **0.238** | **-0.176** |
|  | P.value | 0.000 | 0.068 | 0.000 | 0.000 | 0.002 |
| SLC2A1 | Cor | -0.022 | 0.069 | -0.048 | **-0.116** | -0.008 |
|  | P.value | 0.702 | 0.230 | 0.410 | 0.043 | 0.885 |
| SNCA | Cor | 0.000 | -0.039 | 0.022 | 0.038 | **0.174** |
|  | P.value | 0.995 | 0.497 | 0.699 | 0.507 | 0.002 |
| SPP1 | Cor | **0.301** | **0.354** | **0.455** | **0.720** | **-0.207** |
|  | P.value | 0.000 | 0.000 | 0.000 | 0.000 | 0.000 |
| TRIM22 | Cor | **0.475** | **0.739** | **0.615** | **0.630** | -0.045 |
|  | P.value | 0.000 | 0.000 | 0.000 | 0.000 | 0.438 |
