# Supplementary figures and images for: Model constructions of chemosensitivity and prognosis of high grade serous ovarian cancer based on evaluation of immune microenvironment and immune response
Source: Cancer Cell Int. 2021 Nov 4;21:593. doi: 10.1186/s12935-021-02295-y (PMC8567582; doi:10.1186/s12935-021-02295-y)

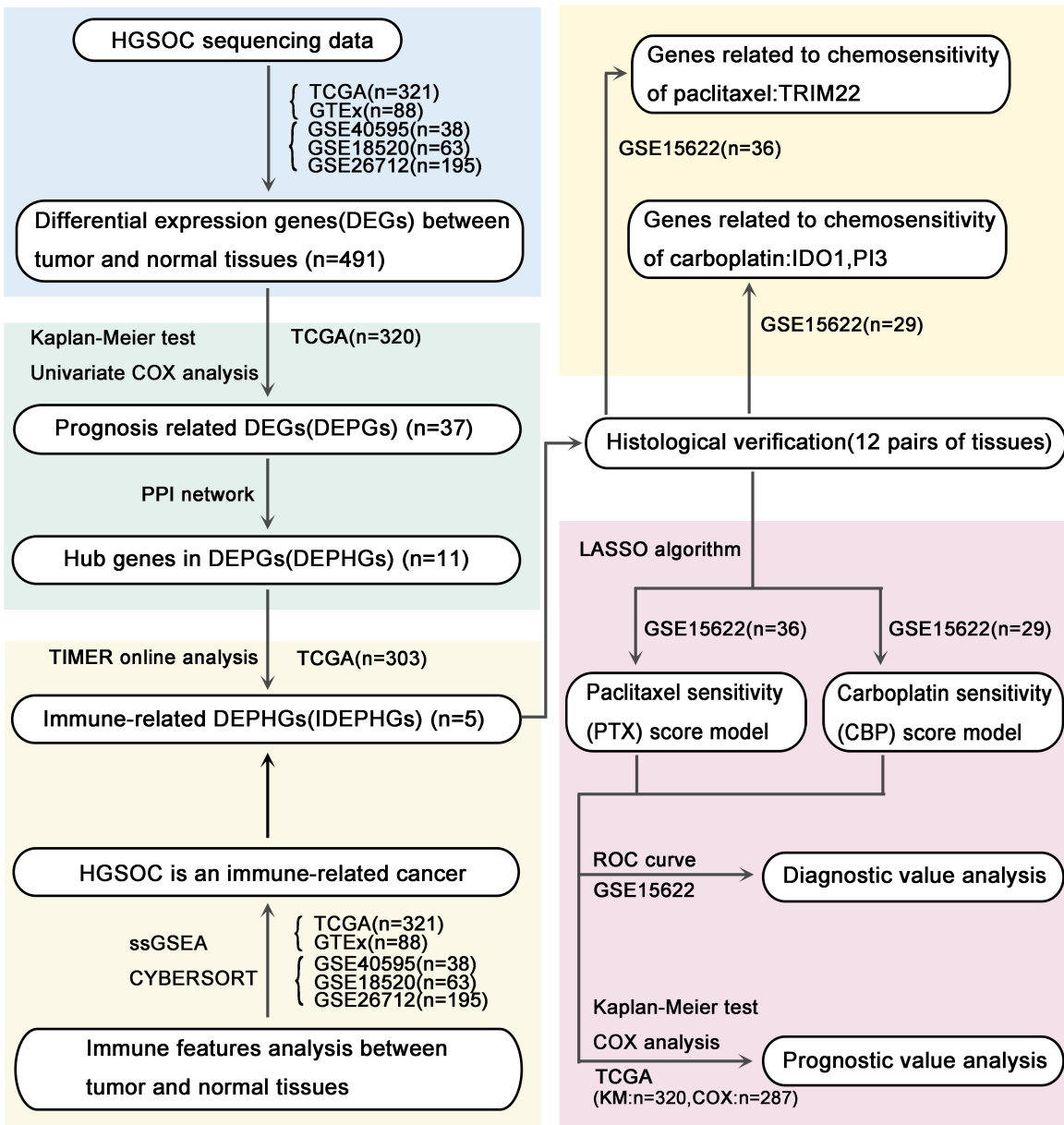

Supplement: Supplementary file 6 — Additional file 6: Fig. S1. A flow chart showing the whole procedures in this study. [file 12935_2021_2295_MOESM6_ESM.pdf]

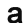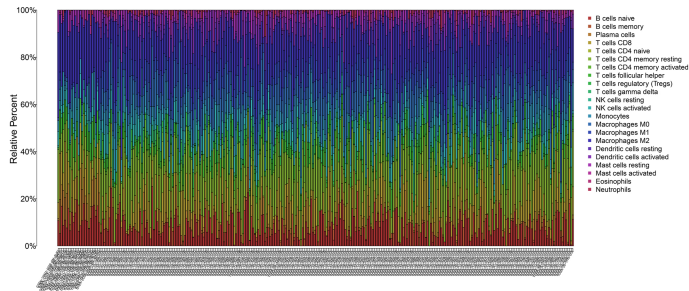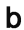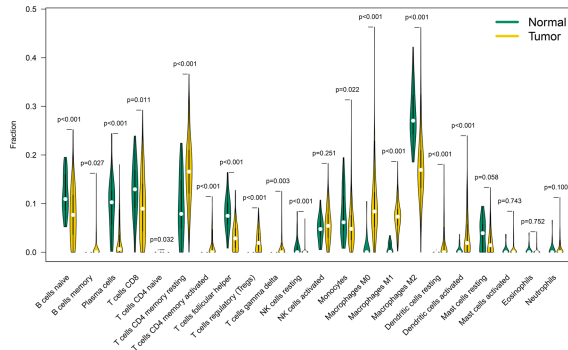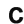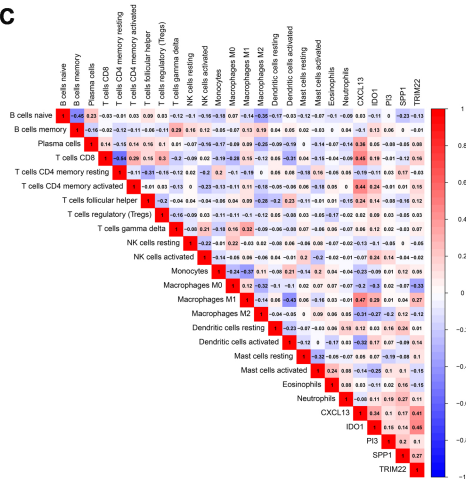

Supplement: Supplementary file 7 — Additional file 7: Fig. S2. CYBERSORT analysis showing the immune characteristics in HGSOC. a. The proportion of 22 kinds of immune cells in each sample. b. Comparation of immune infiltrating cells between normal ovarian epithelium samples (green) and HGSOC samples (yellow) in TCGA+GTEx cohort (tumor tissues form TCGA: n=321, normal tissues from GTEx: n=88). The differences were analyzed by Wilcoxon signed rank test. c. Association between 22 kinds of immune cells and 5 genes expression. Red indicates negative correlation whereas blue indicates positive correlation. Correlation coefficient are labeled on the junction points. [file 12935_2021_2295_MOESM7_ESM.pdf]

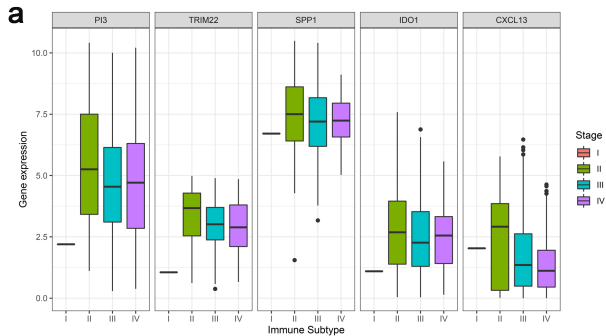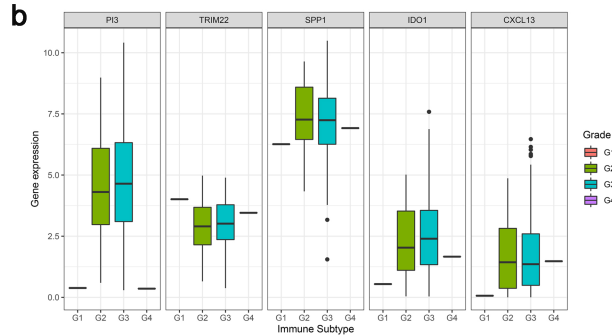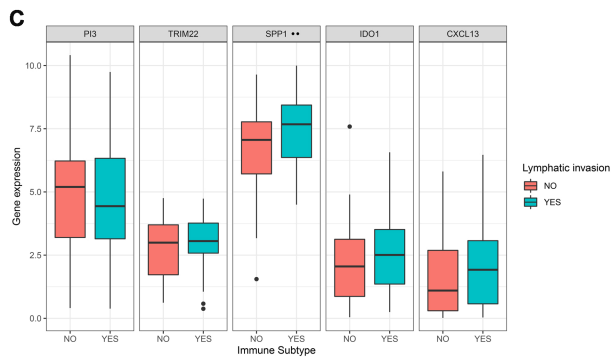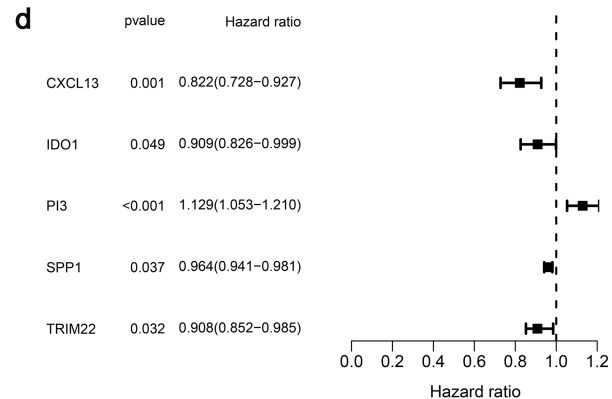

Supplement: Supplementary file 8 — Additional file 8: Fig. S3. Clinicopathological features and prognosis correlation analysis. a-c. Association between genes expression and clinicopathological features in HGSOC. Significance is determined by Wilcoxon rank sum test (*, P<0.05). a. Clinical stage status (I: 1 case; II: 23 cases; III: 295 cases; IV: 57 cases). b. Clinical grade status (G1: 1 case; G2: 45 cases; G3: 322 cases; G4: 1 case). c. Lymphatic invasion (NO: 48 cases; YES: 101 cases). d. COX regression analysis of CXCL13, IDO1, PI3, SPP1 and TRIM22 (n=320). The points represent the HRs, the horizontal line length represents the 95% CI of each group, and the vertical dashed line represents HR=1.0. HR>1 represents a risk factor whereas HR<1 represents a protective factor. [file 12935_2021_2295_MOESM8_ESM.pdf]

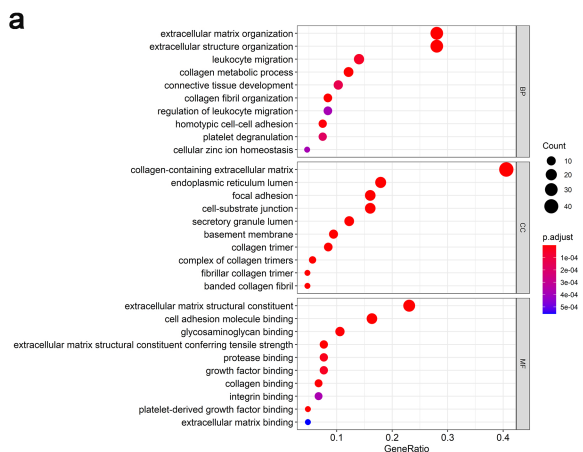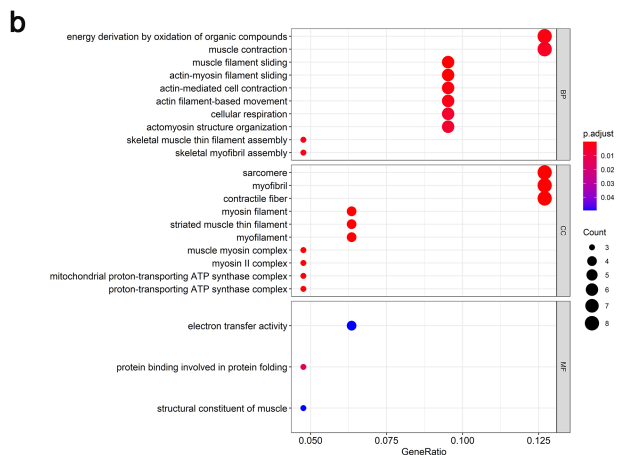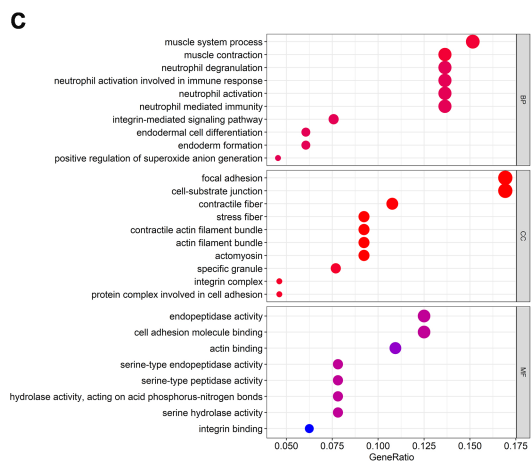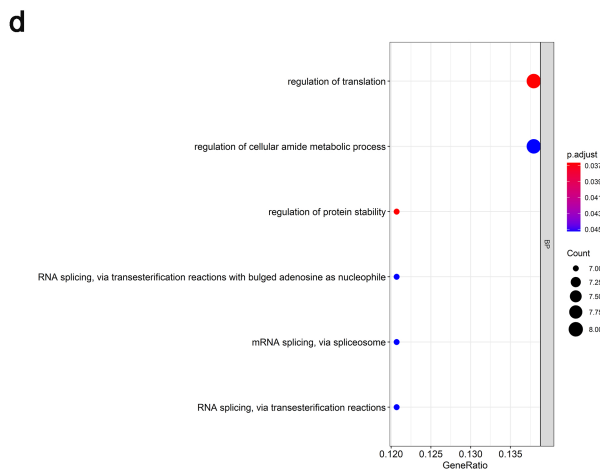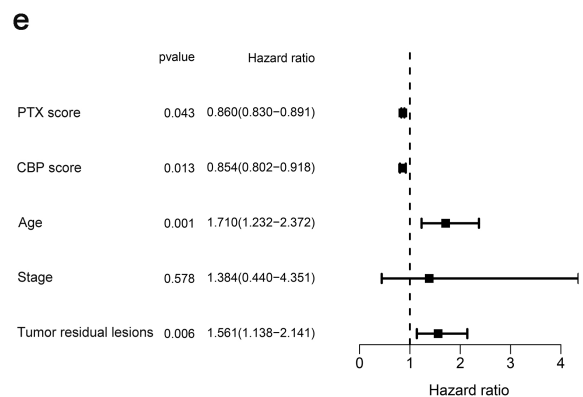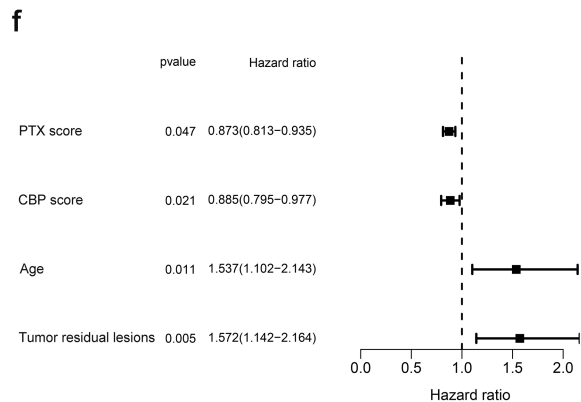

Supplement: Supplementary file 9 — Additional file 9: Fig. S4. GO analysis of GSE15622 and COX regression analysis of TCGA-HGSOC. a-d. GO term enrichment for biological processes (BP), cellular components (CC) and molecular function (MF): a. Paclitaxel sensitive group (n=24). b. Paclitaxel resistant group (n=12). c. Carboplatin sensitive group (n=18). d. Carboplatin resistant group (n=11). e-f. Prognostic value of CBP/PTX score in HGSOC samples from TCGA-OV database (n=287): e. Univariate COX regression analysis showing the prognostic value of age (high group (n=68) ≥ 70 years), stage (high group (n=277) ≥ Stage III), tumor residual lesions (high group (n=81) ≥ 10mm) and CBP/PTX score (high group (n=144) ≥ median value). The points represent the HRs, the horizontal line length represents the 95% CI of each group, and the vertical dashed line represents HR=1.0. f. Multivariate COX regression analysis showing the prognostic value of age, stage, tumor residual lesions and CBP/PTX score. [file 12935_2021_2295_MOESM9_ESM.pdf]
